# Functional characterization of the sugarcane (*Saccharum* spp.) ammonium transporter AMT2;1 suggests a role in ammonium root-to-shoot translocation

**DOI:** 10.3389/fpls.2022.1039041

**Published:** 2022-11-18

**Authors:** Alessandra Koltun, Rodolfo A. Maniero, Marielle Vitti, Nathalia de Setta, Ricardo F. H. Giehl, Joni E. Lima, Antonio Figueira

**Affiliations:** ^1^ Centro de Energia Nuclear na Agricultura, Universidade de São Paulo, Piracicaba, SP, Brazil; ^2^ Centro de Ciências Naturais e Humanas, Universidade Federal do ABC, São Bernardo do Campo, SP, Brazil; ^3^ Instituto de Biociências, Universidade de São Paulo, São Paulo, SP, Brazil; ^4^ Department of Physiology and Cell Biology, Leibniz Institute of Plant Genetics and Crop Plant Research (IPK), Gatersleben, Germany; ^5^ Departamento de Botânica, Universidade Federal de Minas Gerais, Belo Horizonte, MG, Brazil

**Keywords:** ammonium uptake, AMT2 subfamily, nitrogen use efficiency, quadruple mutant, transport kinetics, xylem loading

## Abstract

AMMONIUM TRANSPORTER/METHYLAMMONIUM PERMEASE/RHESUS (AMT) family members transport ammonium across membranes in all life domains. Plant AMTs can be categorized into AMT1 and AMT2 subfamilies. Functional studies of AMTs, particularly AMT1-type, have been conducted using model plants but little is known about the function of AMTs from crops. Sugarcane (*Saccharum* spp.) is a major bioenergy crop that requires heavy nitrogen fertilization but depends on a low carbon-footprint for competitive sustainability. Here, we identified and functionally characterized sugarcane *ScAMT2;1* by complementing ammonium uptake-defective mutants of *Saccharomyces cerevisiae* and *Arabidopsis thaliana*. Reporter gene driven by the *ScAMT2;1* promoter in *A. thaliana* revealed preferential expression in the shoot vasculature and root endodermis/pericycle according to nitrogen availability and source. Arabidopsis quadruple mutant plants expressing *ScAMT2;1* driven by the CaMV35S promoter or by a sugarcane endogenous promoter produced significantly more biomass than mutant plants when grown in NH_4_^+^ and showed more ^15^N-ammonium uptake by roots and nitrogen translocation to shoots. In *A. thaliana*, ScAMT2;1 displayed a K_m_ of 90.17 µM and V_max_ of 338.99 µmoles h^-1^ g^-1^ root DW. Altogether, our results suggest that ScAMT2;1 is a functional high-affinity ammonium transporter that might contribute to ammonium uptake and presumably to root-to-shoot translocation under high NH_4_^+^ conditions.

## Introduction

Nitrogen (N) is the most abundant mineral element present in plant tissues, and nitrate (${\text{NO}}_{3}^{-}$) and ammonium (NH_4_^+^) are the primary inorganic sources absorbed by roots of higher plants. NH_4_^+^ is transported across cell membranes by proteins of the AMMONIUM TRANSPORTER/METHYLAMMONIUM PERMEASE/RHESUS (AMT/MEP/Rh) family (Gazzarrini et al., 1999; Loqué and von Wirén, 2004), which are present in all living organisms (Li et al., 2009; McDonald et al., 2012). Plant AMTs can be further categorized into AMT1 and AMT2 (Loqué and von Wirén, 2004; McDonald et al., 2012). AMT1-type proteins share an evolutionary history related to prokaryotic NH_4_^+^ transporters, while AMT2-type proteins are homologues of the methylammonium permease (MEP) family (von Wittgenstein et al., 2014). The number of AMT family members varies considerably among plant species, displaying a variety of expression patterns, spatial regulations, substrate affinities, and presumed functions (Yuan et al., 2007; Guether et al., 2009; Yuan et al., 2009; McDonald et al., 2012; Li et al., 2016; Giehl et al., 2017; Song et al., 2017).

In *Arabidopsis thaliana*, four root-expressed AMT1 proteins are responsible for high-affinity NH_4_^+^ uptake, namely, AtAMT1;1, AtAMT1;2, AtAMT1;3, and AtAMT1;5 (Loqué et al., 2006; Yuan et al., 2007), while AtAMT1;4 plays a major role in NH_4_^+^ uptake in pollen grains (Yuan et al., 2009). Functional studies revealed that the membrane proteins AtAMT1;1 and AtAMT1;3 are active in the rhizodermis, cortex, and root hairs and are responsible for approximately two-thirds of the NH_4_^+^ uptake capacity by the symplastic route, further supported by AtAMT1;5 in radial transport (Loqué et al., 2006; Yuan et al., 2007). In addition, the apoplastic NH_4_^+^ pool can enter the root symplast by AtAMT1;2 activity in the plasma membrane of endodermal and cortical cells (Yuan et al., 2007). Functional and regulatory characterization of AMT1 proteins in other plant species, including crops (von Wirén et al., 2000; Suenaga et al., 2003; D’Apuzzo et al., 2004; Couturier et al., 2007; Gu et al., 2013; Koegel et al., 2013), have confirmed their dominant role in high-affinity NH_4_^+^ uptake in roots (Loqué et al., 2006; Yuan et al., 2007; Gu et al., 2013).

In contrast to AMT1-type proteins, there is less information about the physiological functions of AMT2 proteins. The sole AMT2 member present in the *A. thaliana* genome, AtAMT2;1, was shown to have a minor role in ammonium uptake under N-deficient conditions (Giehl et al., 2017). However, at elevated N levels, AtAMT2;1 mediates ammonium accumulation in xylem sap and contributes to long-distance ammonium translocation from roots to shoots (Giehl et al., 2017). AMT2 members have been investigated in a few crops, such as wheat (Li et al., 2017b; Jiang et al., 2019), sorghum (Koegel et al., 2013) and maize (Dechorgnat et al., 2019), but various aspects of AMT2 function and regulation remain to be addressed.

Sugarcane (*Saccharum* spp.) is a robust feedstock for bioenergy production due to its remarkably high aboveground biomass, including culms with high sucrose content (Tilman et al., 2009; Waclawovsky et al., 2010; Hoang et al., 2015). High N fertilizer rates are applied to boost sugarcane production; however, the crop responds poorly to N fertilization, and N losses can reach up to 50% (Franco et al., 2008; Robinson et al., 2011). The reason behind the high N loss in sugarcane remains elusive (Thorburn et al., 2017; de Castro et al., 2018; Lima et al., 2022). The low nitrogen use efficiency (NUE) of sugarcane represents high economic and environmental costs (Thorburn et al., 2011; Skocaj et al., 2013), reducing the long-term sustainability of this bioenergy crop (Erisman et al., 2010). Various forms of N losses from the soil (volatilization, N_2_O emission, nitrate leaching, and run-off) negatively impact ecosystems (McAllister et al., 2012). Therefore, it is essential to improve sugarcane NUE to help maintain its competitiveness and sustainability as a bioenergy crop (Thorburn et al., 2017).

NUE is a complex trait involving N uptake, assimilation, and remobilization during plant development (Dobermann, 2005; Li et al., 2017a; Sharma and Bali, 2018). Prospecting genes involved in NUE is challenging, particularly in crops with a highly polyploid and complex genome, such as modern sugarcane cultivars (Thirugnanasambandam et al., 2018). Sugarcane achieves optimal growth and yield under the supply of mixed ammonium and nitrate sources (Otto et al., 2016), yet sugarcane roots have a physiological preference for ammonium over nitrate uptake under N-sufficient (Robinson et al., 2011) or N-limited conditions (Lima et al., 2022). Therefore, the sugarcane preference for NH_4_^+^ may also mean that transporters that are potentially involved in radial NH_4_^+^ transport in roots and/or root-to-shoot NH_4_^+^ translocation may affect the overall NUE of sugarcane, which led us to investigate AMTs to determine the potential role of AMTs in improving NUE in sugarcane. We started by functionally characterizing AMT1-type ammonium transporter members of sugarcane (unpublished results).

In the present work, we searched for AMT2-type ammonium transporters in the sugarcane genome by screening clones from a bacterial artificial chromosome (BAC) library (Tomkins et al., 1999). We then functionally characterized *ScAMT2;1* by complementing ammonium transport-defective mutants of *Saccharomyces cerevisiae* (triple *mep*Δ) and *A. thaliana* (*qko*). The analysis of the *ScAMT2;1* expression profile in response to various N conditions in sugarcane, together with promoter analysis driving a reporter gene, allowed some insight on the regulation of AMT2;1 in NH_4_^+^ transport in response to changes in external N availability and source. Altogether, the evidence suggests a role for AMT2;1 in ammonium uptake and a presumed contribution to root-to-shoot translocation.

## Materials and methods

### Biological materials

AMT2 sequences were searched in a sugarcane BAC library from the commercial cultivar ‘R570’ (Tomkins et al., 1999). Analyses of *ScAMT2;1* expression in sugarcane organs upon various N sources and levels were performed using the commercial cultivar SP80-3280. The *S. cerevisiae* mutant 31019b (triple *mep*Δ: *mep1, mep2::LEU2, mep3::KanMX2, ura3*) (Marini et al., 1997) defective for ammonium uptake was complemented with *AtAMT1;1* or *ScAMT2;1*. The Arabidopsis genotype Columbia-0 (Col-0), the quadruple AMT-knockout mutant *qko* (*amt1;1*, *amt1;2*, *amt1;3*, and *amt2;1*) (Yuan et al., 2007), and the respective complemented lines were used in complementation assays.

### Search for AMT2 and *in silico* analyses of *ScAMT2;1* coding and regulatory regions

Sugarcane *AMTs* were sought in a BAC library that consists of 269 plates with 384 clones each in a total of 103,296 clones representing a 4.5X coverage of the sugarcane genome (Tomkins et al., 1999). The search was performed by real-time PCR amplification of the three-dimensional pool of clones (de Setta et al., 2014). For that, *A. thaliana* and *Oryza sativa AMT2;1* were used to find orthologue sequences in the sugarcane expressed sequence tag (SUCEST) database (https://sucest-fun.org/) to design the primers (**Supplementary Table S1**). First, superpools were screened for positive blocks, and positive blocks were further screened for the specific coordinates of positive clones, which were then isolated for confirmation and sequenced using the 454/Roche sequencing platform, assembled, and automated annotated as previously described (de Setta et al., 2014).

*AMT* gene automated annotation was curated using Artemis Genome Browser and Annotation Tool (v. 16.0.11) (Rutherford et al., 2000), and sorghum *AMT2* was used as a reference. *ScAMTs* were aligned with *AMTs* from maize, rice, sorghum, and *S. spontaneum* by ClustalW (Thompson et al., 2003), including a sugarcane (‘SP80-3280’) *AMT2;1* root-expressed sequence, identified here as ‘comp105883’ (NCBI id# OM966894). The evolutionary history was inferred by using the Maximum Likelihood method and JTT matrix-based model using MEGA11 (Tamura et al., 2021). A discrete Gamma distribution was used to model evolutionary rate differences among sites [5 categories (+*G*, parameter = 1,1177)]. This analysis involved a total of 537 positions in the final amino acid alignment. A physical map of genomic sequences (~100 kb) containing *AMT2;1* from sugarcane (*Saccharum* spp. ‘R570’) BAC clones (032_A12, 038_G02, 118_C18, 216_D16, and 235_F05) and *S. bicolor* (chromosome 9; NC_012878) was manually generated.

*ScAMT2;1* identified in the BAC clones were analyzed to select the sequence to be functionally characterized. Regulatory upstream (~ 3 kb from the start codon) and coding regions were aligned and compared by ClustalW using BioEdit (Hall, 1999). Conceptually translated amino acid sequences were analyzed for specific elements/domains of the MEP/AMT/Rh transporter superfamily using Prosite (Hulo et al., 2006), TMHMM (Krogh et al., 2001), and WebLogo (Crooks et al., 2004). The presence of transposable elements (TEs) in the *ScAMT2;1* regulatory region was predicted by Censor (Kohany et al., 2006) using the Viridiplantae database, and the co-occurrence of transcription factor-binding sites (TFBSs) was analyzed by PlantPAN 2.0 (Chow et al., 2016).

### Sugarcane plant growth and experimental conditions for gene expression analysis

‘SP80-3280’ plantlets derived from *in vitro* meristem culture were grown hydroponically in 5 L plastic pots with full-strength nutrient solution (Hoagland and Arnon, 1950) containing 1 mM NH_4_NO_3_ (pH adjusted to 5.8) under greenhouse conditions for three months. The nutrient solution was aerated and renewed weekly. Prior to treatment, plants received a nutrient solution containing 2 mM NH_4_NO_3_ for 2 d. Subsequently, the plants were subjected to either an N-free nutrient solution (-N), 2 mM NH_4_NO_3_ (+N), 4 mM KNO_3_ (${\text{NO}}_{3}^{-}$), 4 mM NH_4_Cl (${\text{NH}}_{4}^{+}$), or 5 mM NH_4_NO_3_ (high N) for 14 d. Roots, culms, and young (+1) and mature (+3) leaves were collected, frozen in liquid N and stored at -80°C. Three plants per treatment were used for *ScAMT2;1* tissue-specific expression.

### Arabidopsis genotype growth and experimental conditions

Arabidopsis seeds were surface sterilized and grown for 30 d in substrate and vermiculite (1:1) in a growth chamber at 22°C, 80% humidity, and a 16/8 h light/dark phase at 200 µmol m^-2^ s^-1^. For the selection of transgenic events and experiments in agar plates, seeds were sown onto modified half-strength MS with 1 mM NH_4_NO_3_ as the sole N source, with the pH adjusted to 5.8. After a 4 d vernalization at 4°C in the dark, plates were placed in a growth cabinet at 24°C, 16/8 h light/dark phases, and 100 μmol m^-2^ s^-1^. For experiments in agar plates, Arabidopsis seeds were kept for 3 d in half-strength MS medium with 5 mM KNO_3_, with plates positioned vertically. Seedlings were then transferred onto media supplemented with various N sources at the indicated concentrations under the same environmental conditions. Treatments included either 0.5 mM KNO_3_ or 2 mM NH_4_Cl for experiments with plants bearing p35S::*ScAMT2;1* and 2 mM KNO_3_ or 0.2, 2, and 4 mM NH_4_Cl for experiments with *qko*+p2*ScAMT2;1::ScAMT2;1* plants (sugarcane endogenous promoter). After 14 d of treatment, seedlings were harvested, and the dry or fresh weight was measured.

### *AMT2;1* expression analysis by quantitative reverse transcription amplification

Total RNA was isolated from sugarcane leaves as described (Leal et al., 2007) or from Arabidopsis using TRIzol (Thermo Fisher Scientific; Waltham, MS, USA). cDNA was synthesized using SuperScript III Reverse Transcriptase (Thermo Fisher Scientific). Primers were designed based on the *ScAMT2;1* sequence from clone BAC 118_C18 (**Supplementary Table S1**). RT–qPCR was performed with 5 µL of KAPA SYBR FAST (Kapa Biosystems, Wilmington, MA, USA), 0.2 µM of each primer (**Supplementary Table S1**), and 1 µL of diluted cDNA (1:10) in a final volume of 10 µL. Reactions were run in a RotorGene-6000 (Qiagen) with the following settings: 50°C for 10 min and 95°C for 2 min, followed by 40 cycles of 95°C for 20 s, 62°C for 25 s, and 72°C for 25 s. *UBIQUITIN2* was used as a reference gene in sugarcane (*ScUBQ2*) and Arabidopsis (*AtUBQ2*) (**Supplementary Table S1**). All reactions were performed in triplicate with three biological replicates. Relative expression levels were calculated as described (Livak and Schmittgen, 2001). Normalization is indicated for each experiment.

### Functional analysis of ScAMT2;1 by complementation of the yeast triple *mep*Δ mutant

The full-length *ScAMT2;1* coding sequence from clone BAC 118_C18 was synthesized (Biomatik; Cambridge, Ontario, Canada); *AtAMT1;1* was used as a positive control because of its well-established function, and the empty vector was used as a negative control (final constructs in **Supplementary Table S2**). *AtAMT1;1* and *ScAMT2;1* sequences were amplified from Arabidopsis cDNA or synthetic vector, respectively, with primers containing restriction enzyme sites (**Supplementary Table S1**), cloned into pGEM-T Easy (Promega, Madison, WI, USA), sequenced, and subcloned into the expression vector pDR196 (Rentsch et al., 1995). Triple *mep*Δ (strain 31019b) yeast cells were transformed by the lithium acetate method (Gietz and Schiestl, 2007). Confirmed positive clones were inoculated in liquid YNB-AA/AS (0.17% yeast nitrogen base without amino acids or ammonium sulfate) containing 1 mM arginine (positive control) and 50 mg L^-1^ ampicillin for 36 h at 30°C at 200 rpm. A growth test was performed with a serial dilution (DO_600nm_= 1, and subsequent dilution of 10^-1^, 10^-2^, and 10^-3^) plated onto YNB-AA/AS supplemented with 3% glucose and one source of N [0.5, 2, 3, or 5 mM NH_4_Cl (NH_4_^+^), 100 mM methylammonium (MeA), or 1 mM arginine (Arg)]. MES-Tris was added at 20 mM to maintain the pH at 5.0, 6.0, or 7.5. The plates were incubated at 30°C for 6 d.

### Promoter analysis and *ScAMT2;1* functional complementation of the Arabidopsis *qko* mutant

The *ScAMT2;1* promoter region fragment from clones BAC 118_C18 (2,936 bp; p1*ScAMT2;1*) and BAC 235_F05 (2,962 bp; p2*ScAMT2;1*), hereafter called endogenous promoters, as well as the coding region from the synthetic *ScAMT2;1* gene, were amplified (see above). The amplified products were cloned into pDONR or pCR8 (Thermo Fisher Scientific) and then subcloned into the final pMDC vectors (primers in **Supplementary Table S1**; vectors and final constructs in **Supplementary Table S2**) using the Gateway system (Thermo Fisher Scientific). Arabidopsis plants (Col-0 or *qko*) were transformed by floral dipping (Narusaka et al., 2010) using *Agrobacterium tumefaciens* GV3101 bearing the constructs indicated in **Supplementary Table S2**. Transformed lines were selected for hygromycin resistance. Homozygous lines were confirmed by PCR and RT–qPCR.

### Localization of promoter activity in Arabidopsis

Seedlings of Arabidopsis transgenic lines containing the GUS reporter gene (*uidA*, pMDC164) driven by *ScAMT2;1* endogenous promoter were cultivated on half-strength MS media without N (-N) or supplied with 2 mM NH_4_Cl or 1 mM NH_4_NO_3_ as the sole N source for up to 10 d. For GUS staining, plants were transferred to buffer containing 5‐bromo‐4‐chloro‐3‐indolyl glucuronide (X-Gluc; Jersey Lab and Glove Supply, Livingston, NJ, USA) at 37°C for 4 h 30 min and then washed in 70% ethanol (Jefferson et al., 1987). Plant tissues were analyzed and photographed under a Nikon SMZ18 stereo microscope.

### ^15^N uptake and accumulation in Arabidopsis plants

Wild-type Arabidopsis and homozygous (T_3_) *ScAMT2;1*-complemented *qko* plants were hydroponically grown in N-sufficient conditions (2 mM NH_4_NO_3_) with pH adjusted to 5.8 with 2-(N-morpholino)ethanesulfonic acid (MES) for 40 d. For *qko*+*p35S*::*ScAMT2;1* lines, plants were subjected to N deficiency (-N, no N) or N sufficiency (+N, 1 mM NH_4_NO_3_), whereas *qko*+*p2ScAMT2;1::ScAMT2;1* plants were transferred to -N, 2 mM KNO_3_ or NH_4_Cl as sole N sources. After 3 d under treatment, plants were exposed to a short-term ^15^N-ammonium influx assay with a 10 min incubation in a full-strength nutrient solution containing 0.2 mM (^15^NH_4_)_2_SO_4_ (60% of ^15^N-ammonium). To assess ^15^N accumulation in roots and shoots, plants were subjected to -N for 3 d and then transferred to a ^15^N-labelled nutrient solution with 2 mM (^15^NH_4_)_2_SO_4_ (60% of ^15^N-ammonium) for 1 h. For concentration-dependent influx of NH_4_^+^ into roots of *qko* and *qko*+p35S::*ScAMT2;1* lines, 40-d-old plants grown hydroponically under the same conditions mentioned above were transferred to -N for 3 d. Roots were then incubated for 10 min in full nutrient solution containing increasing concentrations 0, 25, 50 100, 150, 200, 300, and 500 mM of (^15^NH_4_)_2_SO_4_ (60% of ^15^N-ammonium). To further assess the contribution of AMT2;1 to ^15^N root-to-shoot translocation, 40-d-old hydroponically grown *qko* and *qko*+ *p2ScAMT2*::*ScAMT2;1* plants were subjected to -N for 3 d followed by one h-root exposure to a nutrient solution containing 0.2 mM or 4 mM (^15^NH_4_)_2_SO_4_ (60% of ^15^N-ammonium). In all ^15^N experiments, roots were first rinsed with 1 mM CaSO_4_ for 1 min before exposure to ^15^N, followed by washing with 1 mM CaSO_4_ prior to sample collection. Roots and shoots were collected separately, dried, ground, and analyzed for total ^15^N content using continuous-flow isotope ratio mass spectrometry (ANCA SL, Sercon, Cheshire, UK).

### Data analysis

A completely randomized design was used in all experiments. The number of biological replicates is indicated for each experiment. Analysis of variance (ANOVA) was performed, and means were compared using Tukey’s test at 5% significance or *t* test (*p* ≤ 0.10 and *p* ≤ 0.05), as indicated for each experiment, using SAS software (SAS Institute Inc., Cary, NC, USA).

## Results

### Identification of *ScAMT2;1* in BAC clones and selection of sequences for functional characterization

Through real-time amplification of the three-dimensional pool of BAC clones using *AMT2;1*-specific primers followed by screening for the specific coordinates of positive clones, we identified five clones containing sequences closely related to *ScAMT2;1* transcript, namely, BAC 032_A12, BAC 038_G02, BAC 118_C18, BAC 216_D16, and BAC 235_F05 (**Supplementary Figure S1**). Each BAC clone contained a unique locus, except for BAC 216_D16 and BAC 235_F05, which shared the same protein sequence. The BAC 032_A12, BAC 038_G02, and BAC 118_C18 *AMT2.1* loci were phylogenetically closer to the root-expressed assembled transcript (comp105883). On the other hand, BAC 216_D16 and BAC 235_F05 did not group closely with the transcript sequence (**Supplementary Figure S1**).

**Figure 1:**
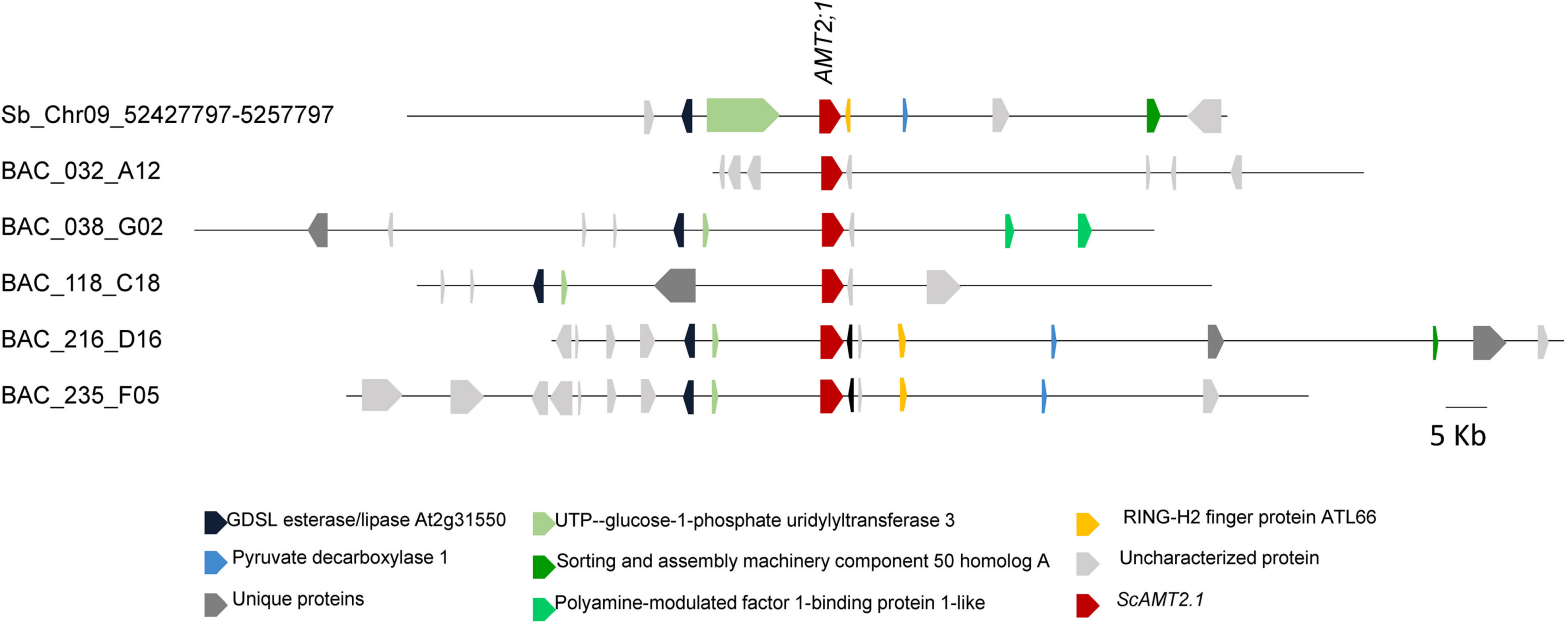
Physical map of genomic sequences (100 Kb) containing *AMT2;1* from sugarcane (*Saccharum* spp. ‘R570’) BAC clones (032_A12, 038_G02, 118_C18, 216_D16, and 235_F05) and *S. bicolor* (chromosome 9; NC_012878).

As sugarcane cultivars are polyploids derived from interspecific crosses between *S. officinarum* and *S. spontaneum* (Nogueira et al., 2005; Graff et al., 2011), the identified BAC clones containing distinct *ScAMT2;1* loci are expected to differ for the surrounding topology (**Figure 1**). Of the five clones, BAC 216_D16 and BAC 235_F05 were highly similar, with 62.0% identity.

The alignment of the deduced amino acid of AMT2;1 sequences from the BAC clones, comp105883, and one *S. spontaneum AMT2;1* (Wu et al., 2021) indicated that BAC 118_C18 had an identical protein sequence as the transcribed assembled sequence (comp105883) (**Supplementary Figure S3A**). The complete *ScAMT2;1* gene from BAC 118_C18 (NCBI# OM471796) is 1,473 bp long with three exons encoding 490 amino acids, conceptually estimated to be a protein of 52 kDa (**Supplementary Figure S2B**). Similar gene structure and protein features were observed for *S. spontaneum SsAMT2;1* from the chromosome 7 (Sspon.03G0003380-4D; Wu et al., 2021) (**Supplementary Figure S2B**; **Supplementary Figure S3A**). In addition, the presumed ScAMT2;1 amino acid sequence from BAC 118_C18 was identical to the SsAMT2;1 protein Sspon.03G0003380-4D (**Supplementary Figure S3A**) and contained the expected 11 transmembrane domains predicted by TMHMM (**Supplementary Figure S3B**), along with the conserved signature motif for the MEP/AMT/Rh superfamily (**Supplementary Figure S3C**).

Various plant AMT1 and AMT2 sequences, including the identified sugarcane members, were compared to verify the conservation of C- and N-terminal regions concerning amino acids essential for transport function (**Supplementary Figure S4**). In the N-terminus of the tomato protein LeAMT1;1, two cysteines (C3 and C27) have been proven to be fundamental for AMT1 oligomer stability (Graff et al., 2011). While AMT1 proteins except for SlAMT1;3 contained these two conserved Cys residues, these residues were absent in all AMT2 homologues (**Supplementary Figures S4A, C**). In the C-terminus, some residues have been associated with transport regulation, including glycine-456 (G456; SlAMT1;1) and threonine-460 (T460) (Ludewig et al., 2003). G456 was found in all AMTs evaluated to date, whereas T460 was absent in all AMT2 subfamily members, including ScATM2;1 (**Supplementary Figures S4B, D**).

Multiple alignment of the various *ScAMT2;1* promoter sequences identified in the BAC clones (approximately 3 kb upstream of the predicted translation start codon) allowed the arbitrary separation of the clones into two groups, in which sequences from BAC 032_A12, BAC 038_G02, and BAC 118_C18 were more similar between each other, differing from BAC 216_D16 and BAC 235_F05 (**Supplementary Figure S5A**). We analyzed whether this separation could be due to transposable element (TE) insertions, which are commonly found in promoter regions of sugarcane sequences (de Setta et al., 2014). TE insertion was assessed by Censor, which identified repetitive elements by comparison with known repeats and assigned a score for probability. The results reinforced the similarity of BAC 216_D16 and BAC 235_F05, showing a similar TE insertion profile (**Supplementary Figure S5C**). To further investigate the presence of regulatory elements and presumed synteny of regulatory motifs, we chose clones from each group, BAC 118_C18 and BAC 235_F05. Only a few conserved regions exist between the selected regulatory regions, indicating significant variation between the two *ScAMT2;1* promoters (**Supplementary Figure S5C**). As loci from BAC 118_C18 were not functional in driving the expression of *uidA* in the GUS assay (see below), the sequence from the BAC 235_F05 clone was chosen to be further analyzed as a functional *ScAMT2;1* endogenous promoter. Concerning the gene sequence, *ScAMT2;1* from BAC 118_C18 was selected for functional validation due to greater similarity with the root-expressed *ScAMT2;1* sequence comp105883 and *S. spontaneum* Sspon.03G0003380-4D (**Supplementary Figure S2B**).

### *ScAMT2;1* is expressed in sugarcane roots and shoots and it is regulated according to inorganic N source and level

**Figure 2:**
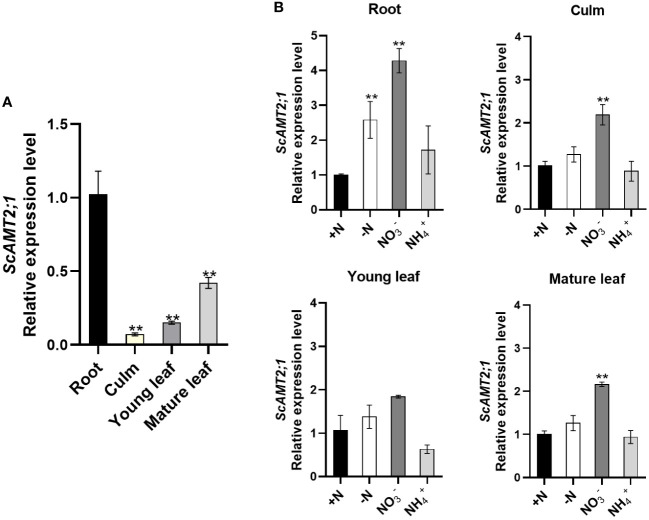
Expression of *ScAMT2;1* in sugarcane organs after subjecting plants to distinct inorganic N sources or no N. **(A)** RT–qPCR analysis of *ScAMT2;1* expression in roots, culms, and young (+1) or mature (+3) leaves of sugarcane grown under 2 mM NH_4_NO_3_ for 2 d. Bars indicate ± SE (*n* = 3). *ScUBQ2* was adopted as a reference gene. Gene expression levels were normalized to expression levels in culms. Asterisks represent significant differences in relation to culms according to Student’s *t* test (*p* < 0.01). **(B)** *ScAMT2;1* relative expression levels in sugarcane roots, culm, young, and mature leaves of plants under +N: 2 mM NH_4_NO_3_, -N: no N; ${\text{NO}}_{3}^{-}$: 4 mM KNO_3_, or NH_4_^+^: 4 mM NH_4_Cl for 14 d. *ScUBQ2* was used as a reference gene. The gene expression level was normalized to the +N treatment. Bars indicate ± SE (*n* = 3). Asterisks represent significant differences between treatments and +N according to Student’s *t* test (*p* < 0.01).

The transcriptional profile of *ScAMT2;1* was examined in the organs of sugarcane plants grown under various N conditions. At the stage of generative growth (90-d-old plants) under N-sufficient conditions, *ScAMT2;1* was expressed in all organs analyzed, with more transcript accumulation in roots, but it was also largely expressed in mature leaves, followed by young leaves, and less abundant in culms (**Figure 2A**). To assess how *ScAMT2;1* expression is regulated by N supply, transcript levels were determined in various organs in plants grown in nutrient solution containing distinct N sources or without N for 14 d and compared with the +N treatment (**Figure 2B**). In the presence of nitrate as the sole N source, *ScAMT2;1* transcripts accumulated approximately 2- to 3-fold more in roots, mature leaves, and culms but not in young leaves compared with plants grown in ammonium nitrate (+N). Thus, exposing the plants at the same high N level (4 mM) but changing the source from 2 mM NH_4_NO_3_ to 4 mM KNO_3_ (4 mM N with no ammonium) was sufficient to induce *ScAMT2;1* expression in roots and shoots more than the change from 2 mM NH_4_NO_3_ to 4 mM NH_4_Cl.

To further investigate whether *ScAMT2;1* is transcriptionally modulated by N availability, the expression profile was evaluated in sugarcane plants grown under 5 mM NH_4_NO_3_ (high N) or no N (-N) for 10 d (**Supplementary Figure S6**). Transient and temporal transcript accumulation was detected in N-deficient mature leaves relative to high N supply. In culms, *ScAMT2;1* transcripts showed some accumulation in both treatments; however, this transcriptional response was not observed in roots or young leaves. Altogether, these observations indicate that the N source and the plant N status modulate the expression of *ScAMT2;1* in sugarcane.

### *ScAMT2;1* marginally facilitates ammonium uptake in a defective mutant yeast

**Figure 3:**
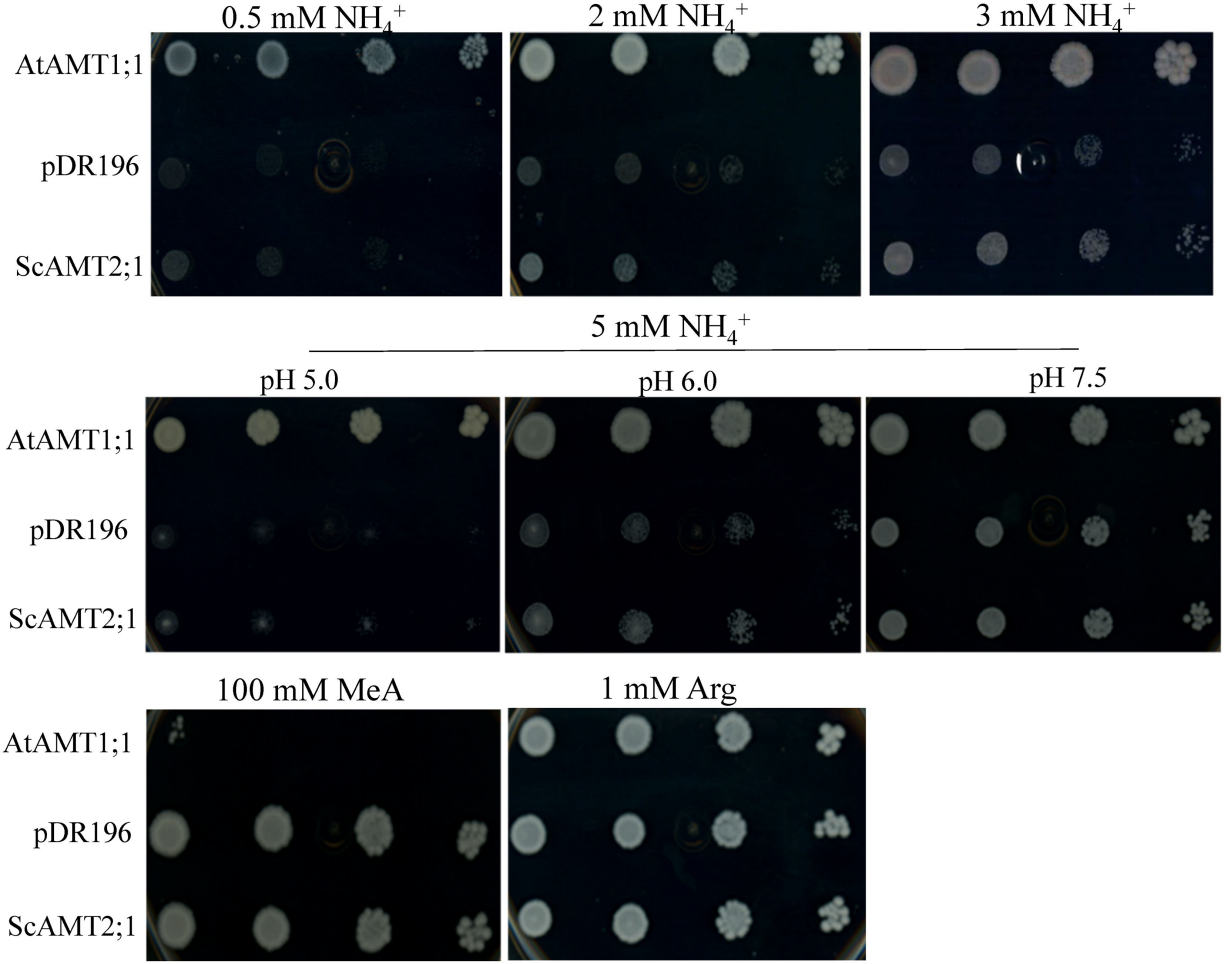
Functional complementation of the yeast mutant defective for ammonium uptake. Growth of the ammonium transporter-deficient yeast strain triple *mep*Δ (31019b) expressing *AtAMT1;1* (positive control), the empty pDR196 vector (negative control), or *ScAMT2;1* on media supplied with 0.5 to 5 mM ammonium chloride (NH_4_^+^), 100 mM methylammonium (MeA), or 1 mM arginine (Arg; positive control) as the sole N source. Culture media pH was adjusted to 6.0 when not indicated otherwise. Each transformant line was grown to OD_600nm_ = 1 and plated in concentrated and sequential four 10-fold dilutions.

To investigate whether the selected *ScAMT2;1* gene (BAC118_C18) encodes a functional ammonium transporter, we complemented the *S. cerevisiae* triple *mep* mutant (31019b) (Marini et al., 1997). The positive control (triple *mep* complemented with AtAMT1;1) completely restored growth under all N conditions tested (**Figure 3**). By increasing the external NH_4_^+^ concentration, triple *mep* cells complemented with *ScAMT2;1* showed slightly more growth than the negative control suggesting that ScAMT2;1 is a functional protein that mediates ammonium transport. At 5 mM ammonium, the growth of the triple *mep* complemented with ScAMT2;1 was strongly pH dependent. The ScAMT2;1-expressing triple *mep* grew slightly better than the negative control (empty pDR196) at a pH of 5.0 and 6.0. Raising the pH further to 7.5 may have increased the concentration of ammonia (NH_3_), resulting in similar growth between triple *mep* expressing ScAMT2;1 and the negative control.

In contrast to type 1 AMT proteins, AMT2 has been proposed to be impermeable to the transport of the ammonium toxic analogue methylammonium (MeA) (Sohlenkamp et al., 2000; Sohlenkamp et al., 2002). The growth of triple *mep* complemented with ScAMT2;1 was evaluated on media supplemented with 100 mM MeA (**Figure 3**). The toxic effect of MeA drastically reduced the growth of triple *mep* cells expressing AtAMT1;1, whereas those complemented with ScAMT2;1 or the empty vector displayed no visible sensitivity towards MeA.

### *ScAMT2;1* complements the ammonium uptake-defective Arabidopsis quadruple mutant

**Figure 4:**
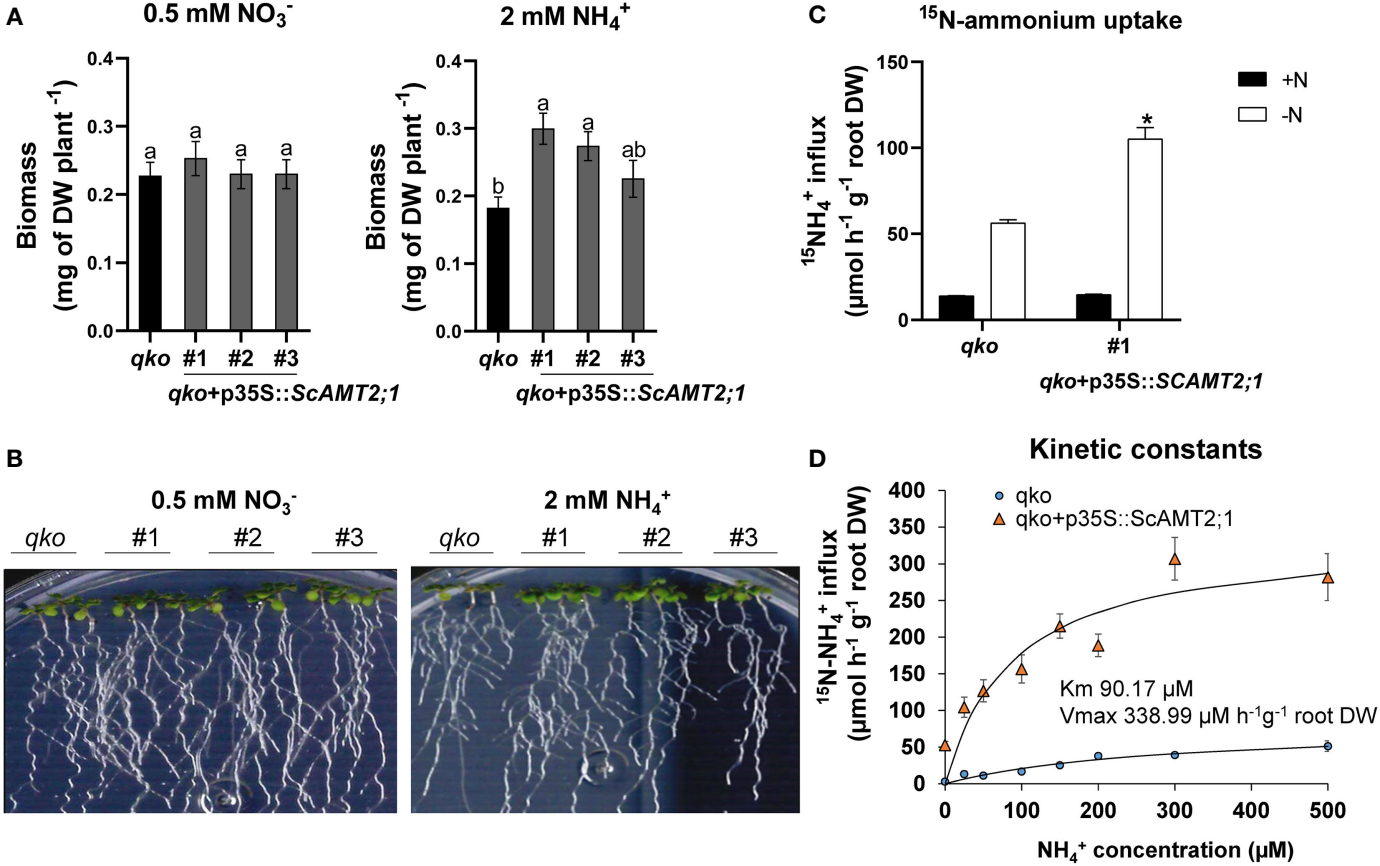
Functional evaluation of complemented Arabidopsis *qko* mutant. **(A)** Total biomass (DW, dry weight) of homozygous lines of Arabidopsis overexpressing *ScAMT2;1* (*qko*+p35S::*ScAMT2;1*) subjected to either 0.5 mM KNO_3_ (${\text{NO}}_{3}^{-}$) or 2 mM NH_4_Cl (NH_4_^+^) for 14 d. Bars indicate means ± SE (*n* = 6). Different letters indicate significant differences among means according to Tukey’s test (*p* ≤ 0.05). **(B)** Phenotype of *qko* and transgenic events grown *in vitro* under different N sources. **(C)** Influx of ^15^N-labeled ammonium into the roots of *qko* and a transgenic line (#1) overexpressing *ScAMT2;1* subjected to either N-free (-N) or 0.2 mM NH_4_^+^ (+N) nutrient solution for 3 d. Bars indicate means ± SE (*n* = 4). Asterisks indicate significant differences between *qko* and the transgenic line according to Student’s *t* test (*p* < 0.05). **(D)** Concentration-dependent influx of ^15^NH_4_^+^ into roots of *qko* or *qko*+p35S::*ScAMT2;1* (#1). Symbols indicate six biological replicates (*n* = 6).

*ScAMT2;1* driven by the CaMV*35S* promoter (p35S) was expressed in the Arabidopsis quadruple *AMT* mutant line (*qko*) (Yuan et al., 2007). Three independent T_3_ homozygous lines were characterized for *ScAMT2;1* expression in relation to Col-0 plants (**Supplementary Figure S7**) and then used for phenotypic evaluation. *ScAMT2;1-*complemented events grown in the presence of ammonium as the only N source accumulated significantly more total dry biomass than *qko*, with values approximately 65% (event #1) and 51% (event #2) higher under 2 mM NH_4_^+^ (**Figures 4A, B**). In contrast, no significant difference between *qko* and the complemented lines was observed when only nitrate was supplied (**Figures 4A, B**), suggesting that the ectopic expression of *ScAMT2;1* restored the *qko* mutant growth phenotype only under ammonium nutrition, likely by mediating ammonium uptake into roots.

We then evaluated the short-term influx of ^15^N-NH_4_^+^ in the *qko ScAMT2;1*-complemented lines. Under -N, the root ammonium uptake capacity of *qko*+p35S::*ScAMT2;1* increased by 87% compared with *qko* (**Figure 4C**), corroborating the function of ScAMT2;1 in NH_4_^+^ uptake in roots. To estimate the substrate affinity of ScAMT2;1, six-week-old *ScAMT2;1-*overexpressing (p35S) *qko* plants were grown under -N for 3 d, followed by concentration-dependent ^15^N-NH_4_^+^ influx analyses. In this experiment, ScAMT2;1 function was saturated above 90 µM (**Figure 4D**). The estimated net ammonium influx fitted the Michaelis–Menten equation well, resulting in a K_m_ = 90.17 µM and a V_max_ of 338.99 µmoles h^-1^ g^-1^ root DW, determined by subtracting the values of *qko* (**Figure 4D**). These results demonstrate that the ScAMT2;1 protein can contribute to high-affinity ammonium transport *in planta*.

### *ScAMT2;1* regulatory region drives expression in Arabidopsis root and shoot vascular tissues and is regulated by N source and availability

**Figure 5:**
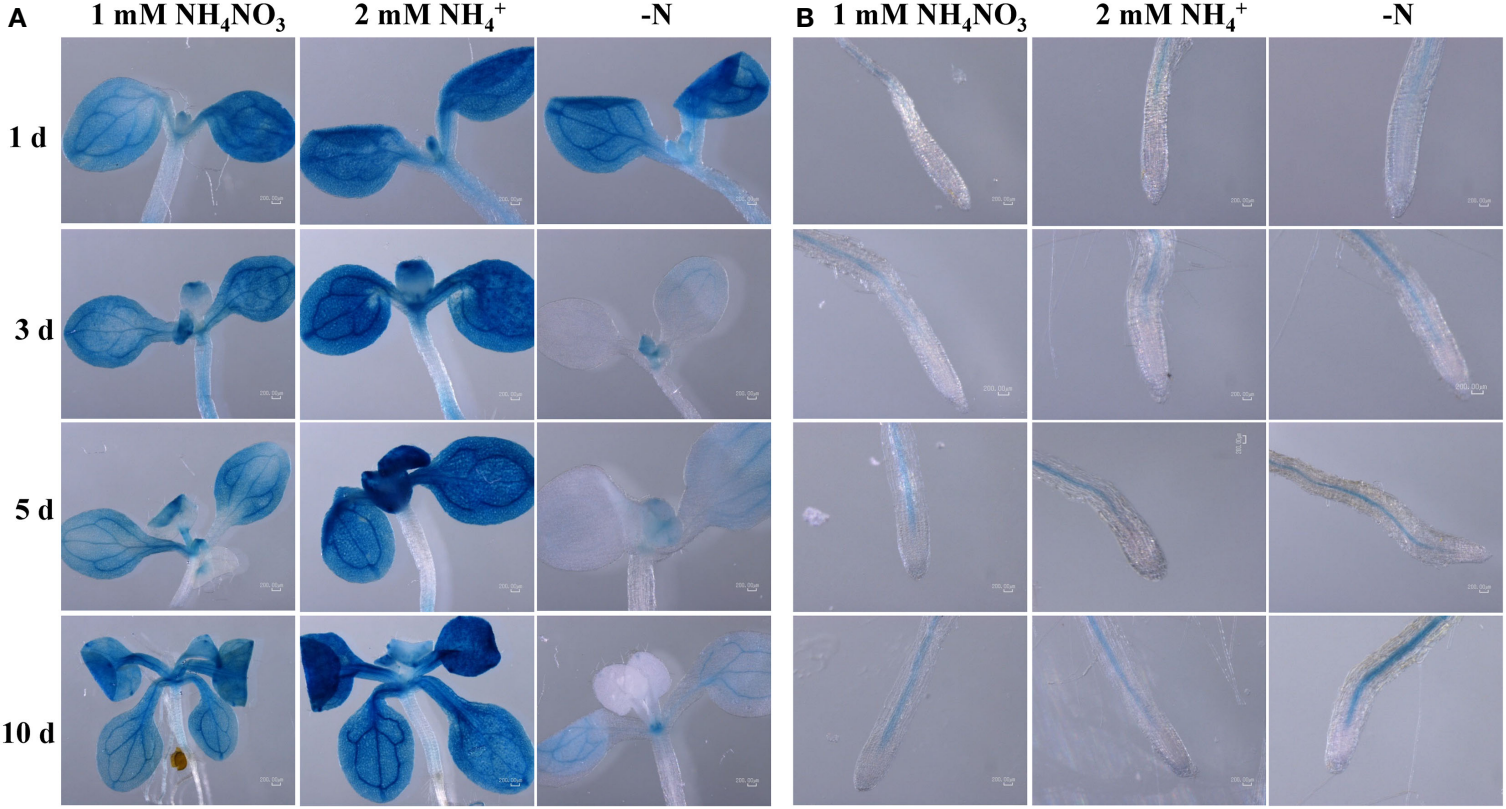
Endogenous *ScAMT2;1* promoter **(**p2**)** driving GUS expression in Col-0 Arabidopsis plants subjected to 1 mM NH_4_NO_3_, 2 mM NH_4_^+^, or no N for 1, 3, 5, and 10 d in **(A)** shoots; and **(B)** roots. The blue color shows GUS activity. Bars = 200 μm.

To help determining the ScAMT2;1 function, we conducted localization experiments in Arabidopsis by expressing the *GUS* reporter gene driven by the *ScAMT2;1* regulatory region from BAC 118_C18 (p1*ScAMT2;1*) and BAC 235_F05 (p2*ScAMT2;1*). No reporter expression was detected with the promoter p1*ScAMT2;1*, which was apparently nonfunctional (**Supplementary Figure S8**). Arabidopsis lines expressing p2*ScAMT2;1*::*GUS* allowed tracing promoter activity in vascular bundles and outermost cells in leaves, either under ammonium or ammonium nitrate (**Figure 5A**). In contrast, leaves from N-deficient plants displayed no p2*ScAMT2;1* activity in outer cells, and activity appeared to predominate at vascular bundles (**Figure 5A**). In roots, GUS was mainly detected in the innermost tissues (**Figure 5B**). Altogether, these results suggest that the *ScAMT2;1* regulatory region is associated with root and leaf vascular tissues, but tissue-specific expression depends particularly on the N status in leaves rather than roots.

### Sugarcane *ScAMT2;1* promoter regulates ammonium uptake according to external N level and source

**Figure 6:**
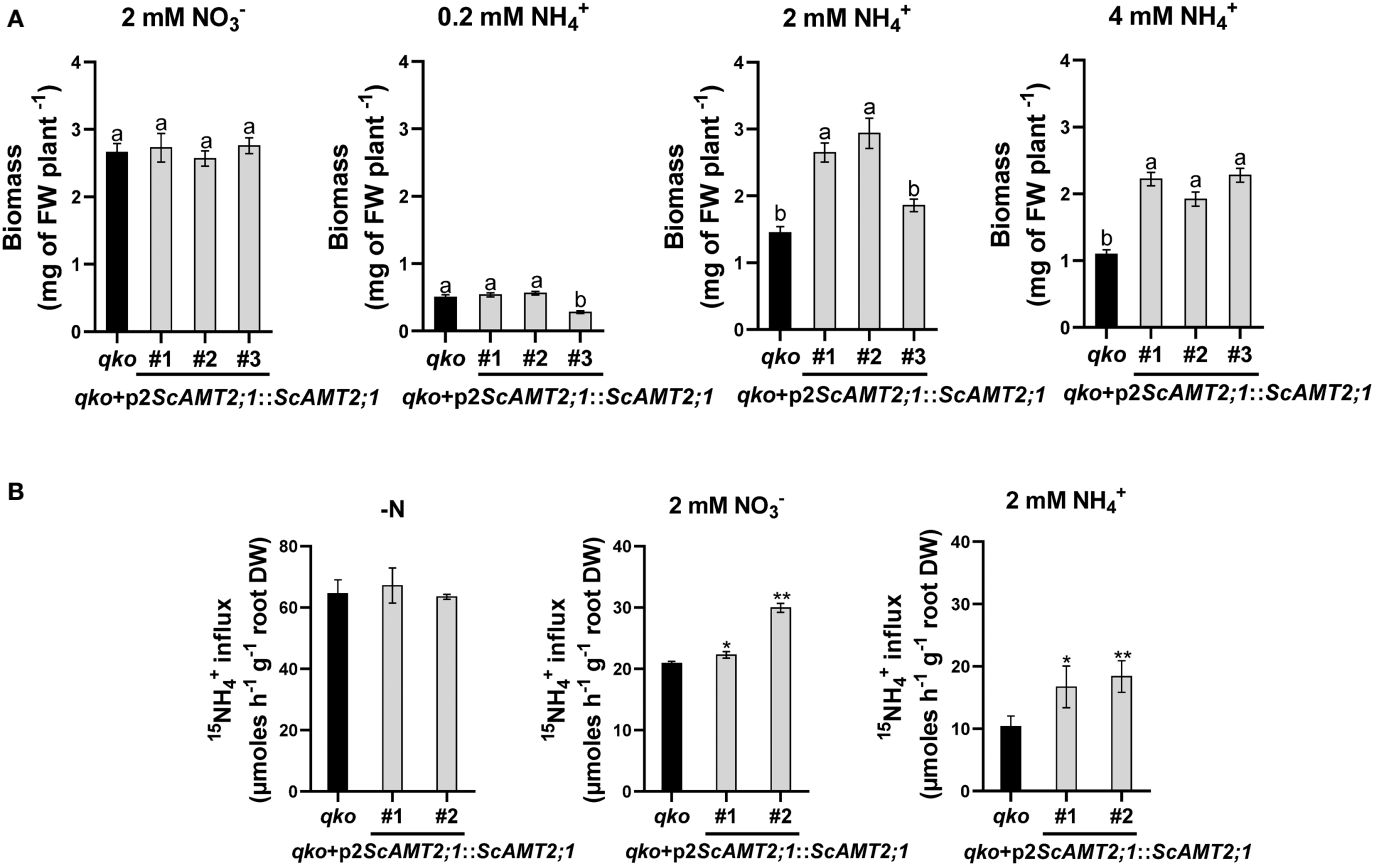
Biomass accumulation in Arabidopsis mutant plants and lines complemented with *ScAMT2;1* driven by the sugarcane endogenous promoter. **(A)** Fresh weight of homozygous lines of *qko*+p2*ScAMT2;1*::*ScAMT2;1* subjected to 2 mM KNO_3_ (${\text{NO}}_{3}^{-}$) or 0.2, 2, and 4 mM NH_4_Cl (NH_4_^+^) for 14 d. Bars indicate means ± SE (*n* = 30). Different letters indicate significant differences among means according to Tukey’s test (*p* ≤ 0.05). **(B)** Influx of ^15^N-labeled ammonium (NH_4_^+^) into roots of *qko* and *qko*+p2*ScAMT2;1*::*ScAMT2;1* lines upon 3-d exposure to N-free (-N), 2 mM ${\text{NO}}_{3}^{-}$, or 2 mM NH_4_^+^ nutrient solution. Bars indicate means ± SE (*n* = 4). Asterisks indicate significant differences between *qko* and transgenic plants according to Student’s *t* test (*p < 0.10 and **p < 0.05).

We then assessed the contribution of ScAMT2;1 to ammonium uptake by generating *qko* lines complemented with *ScAMT2;1* driven by the endogenous regulatory region p2*ScAMT2;1*. While all p2*ScAMT2;1*::*ScAMT2;1-*complemented lines and *qko* grew similarly on agar medium supplemented with either 2 mM nitrate or 0.2 mM ammonium (**Figure 6A**), the total biomass of *qko*+p2*ScAMT2;1*::*ScAMT2;1* plants was clearly superior to that of *qko* under higher external NH_4_^+^ concentrations. At 2 mM NH_4_^+^, *qko*+p2*ScAMT2;1*::*ScAMT2;1* accumulated approximately 83% (#1), 103% (#2), or 28% (#3) more shoot biomass than *qko* (**Figure 6A**). The biomass accumulation for plants grown at 4 mM NH_4_^+^ was 102% (event #1), 75% (event #2), or 107% (event #3) higher than the control plants (**Figure 6A**). These results suggest that ScAMT2;1 under the control of the sugarcane endogenous promoter significantly increased biomass at elevated external ammonium levels and confirm the functionality of ScAMT2;1 in facilitating NH_4_^+^ uptake.

To evaluate the regulatory level of the response of the *ScAMT2;1* promoter to high external N supply, short-term ^15^N-ammonium influx analysis was performed with *qko+*p2*ScAMT2;1*::*ScAMT2;1* plants in the presence of 2 mM of either ammonium or nitrate or no N (-N). The influx of ^15^N-NH_4_^+^ into the roots of *qko* and complemented lines subjected to -N was similar and nonsignificant (**Figure 6B**). Complementation with *ScAMT2;1* significantly increased uptake levels by 6% (#1) and 43% (#2) compared with *qko* when subjected to 2 mM nitrate and by 61% (#1) and 78% (#2) in complemented plants subjected to 2 mM ammonium (**Figure 6B**). Altogether, these results indicate that the regulation of ScAMT2;1 in ammonium uptake depends strictly on the preconditioning of plants to an externally high N form but not to -N.

### The *ScAMT2;1* regulatory region appears to drive ammonium root-to-shoot translocation

**Figure 7:**
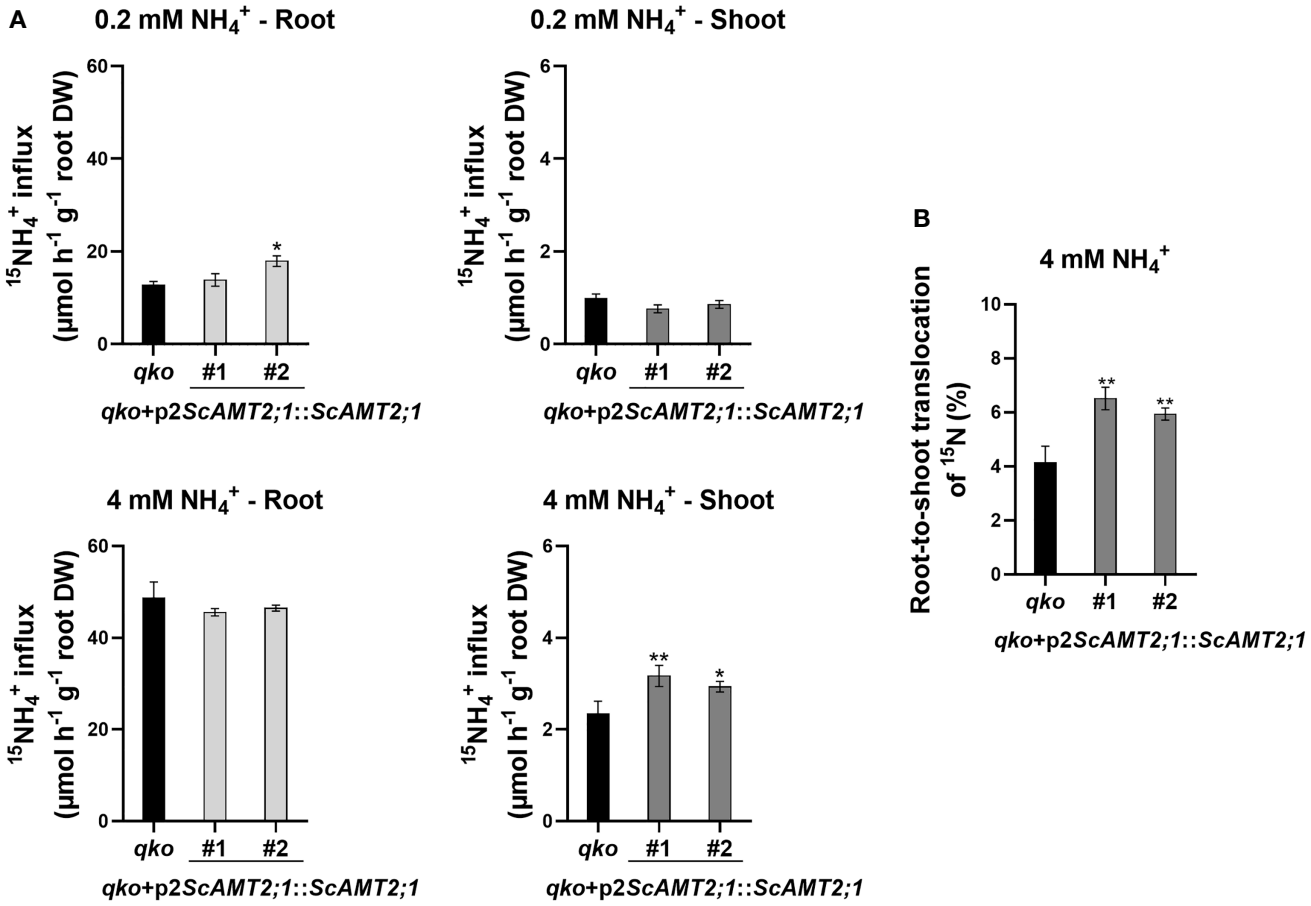
^15^N accumulation in roots and shoots of *qko* and complemented *qko* transgenic lines expressing *ScAMT2;1* under the regulation of its sugarcane endogenous promoter (p2). The assay was performed in plants after 3 d of exposure to an N-free nutrient solution, followed by 1 h of treatment in either a 0.2 mM or 4.0 mM ^15^NH_4_^+^ solution. Bars indicate means ± SE (*n* = 5). Asterisks indicate significant differences in root and shoot ^15^NH_4_^+^ accumulation between *qko* and transgenic plants according to Student’s *t* test (*p < 0.1 and **p < 0.05). **(B)** Root-to-shoot translocation of ^15^N in percent of ^15^N accumulation in shoots in relation to the whole plant. Asterisks indicate significant differences between *qko* and transgenic plants according to Student’s *t* test (** *p* < 0.05).

Our experiments indicated that ScAMT2;1 contributes to root ammonium uptake mainly in NH_4_^+^-supplied plants (**Figure 6**) and that the *ScAMT2;1* promoter (p2) drives gene expression in the inner vascular root cells (**Figure 5**). These results prompted us to evaluate whether ScAMT2;1 mediates root-to-shoot ammonium transport under ammonium supply. To this end, we evaluated ScAMT2;1-specific functions by estimating ^15^N accumulation in roots and shoots of *qko* and *qko+*p2*ScAMT2;1*::*ScAMT2;1* lines subjected to either 0.2 mM or 4 mM ^15^N-NH_4_^+^ for 1 h to allow time for root-to-shoot translocation (**Figure 7A**). At 0.2 mM ^15^N-NH_4_^+^ supply, no significant ^15^N was accumulated in shoots compared with *qko*, whereas some ^15^N accumulation in roots occurred for one transgenic line (event #2). When plants were grown in the presence of 4 mM ^15^N-NH_4_^+^, roots of *qko* and complemented lines accumulated ^15^N in a similar pattern. In contrast, significantly more ^15^N accumulated in the shoots of both *qko*+p2*ScAMT2;1*::*ScAMT2;1* lines, approximately 35% and 25% more than in *qko* shoots (**Figure 7A**). The rate of ^15^N accumulated in the shoot in relation to the whole plant was 7 and 6.3% for the *qko+*p2*ScAMT2;1*::*ScAMT2;1* lines, significantly superior to the 4.8% observed in *qko* when plants were subjected to 4 mM ^15^N-NH_4_^+^ (**Figure 7B**). These results suggest that ScAMT2;1 activity in roots might contribute to ammonium translocation to shoots under high external ammonium conditions.

## Discussion

AMT2 proteins have been demonstrated to exhibit transport properties distinct from those of the AMT1 subfamily (Sohlenkamp et al., 2002; Mayer and Ludewig, 2006; Neuhäuser et al., 2009). However, little is known about the physiological roles of AMT2-type ammonium transporters. In *Arabidopsis thaliana*, the only AMT2 member was shown to play a critical role in root-to-shoot partitioning of ammonium (Giehl et al., 2017). Here, we provide evidence that ScAMT2;1 might be involved in sugarcane root ammonium uptake at elevated external substrate levels.

We started by identifying AMT2s in the sugarcane genome by searching a BAC library from the commercial cultivar R570 (Tomkins et al., 1999). The screening allowed the identification of five *ScAMT2;1* sequences. Modern sugarcane cultivars are derived from interspecific crosses between *S. officinarum* and *S. spontaneum*, followed by backcrossing to *S. officinarum*, producing a highly complex genome (Thirugnanasambandam et al., 2018). Therefore, considering the high polyploidy and redundant character of the sugarcane genome (Garsmeur et al., 2018; Zhang et al., 2018), a series of *in silico* conceptual analyses was performed with the various upstream regulatory regions and gene sequences of the *ScAMT2;1* loci found in the five BAC clones to define the target for functional characterization. Based on the phylogenetic analysis, it was possible to infer that BAC 216_D16 and BAC 235_F05 clones contained *ScAMT2;1* alleles distinct from BAC 038_G02, BAC 032_A12, and BAC 118_C18, which was corroborated by analyzing their regulatory regions. The *ScAMT2;1* coding sequence from BAC 118_C18 was chosen because it demonstrated the highest similarity to a root-expressed sequence detected by us in sugarcane (NCBI# OM966894). In addition, *ScAMT2;1* from BAC 118_C18 was structurally identical to an *AMT2;1* from *S. spontaneum* (Sspon.03G0003380-4D; **Supplementary Figure S2**), with an identical deduced protein (**Supplementary Figure S3A**). The conceptually translated ScAMT2;1 protein from clone BAC 118_C18 displays the conserved 11 presumed transmembrane domains, amino and carboxy-terminal, facing the inner and outer parts of the plasma membrane, respectively, and contains the superfamily signature motif, strongly indicating that this gene is an ammonium transporter (**Supplementary Figure S2**) (Marini et al., 1997; Marini and André, 2000; Schwacke et al., 2003; Loqué and von Wirén, 2004; Ellerbeck et al., 2013). It will be necessary to evaluate whether the other ScAMT2;1 alleles are functional and present the same expression and regulation patterns and transport characteristics as the one evaluated here.

*ScAMT2;1* expression in sugarcane was more pronounced in roots and, to a minor extent, in aboveground organs, especially mature leaves (**Figure 2A**). Similar patterns were found for *AMT2;1* expression in *A. thaliana* (Sohlenkamp et al., 2002), *O. sativa* (Suenaga et al., 2003), *Lotus japonicus* (Simon-Rosin et al., 2003), and *S. bicolor* (Koegel et al., 2013), in all cases showing expression in distinct plant organs. For instance, the homologue *PbAMT2* from *Pyrus betulaefolia* was shown to be expressed in stems, petioles, and leaves but primarily in roots (Li et al., 2016), similar to the *ScAMT2;1* expression pattern described here for sugarcane. Conversely, *PtAMT2;1* from *P. trichocarpa* was shown to be exclusively expressed in roots (Couturier et al., 2007). Analysis of the expression profile in sugarcane roots showed that *ScAMT2;1* transcription was slightly but significantly induced by the N status and significantly induced by ${\text{NO}}_{3}^{-}$ (possibly sensing the lack of NH_4_^+^) in roots, culms, and mature leaves (**Figure 2B**). In Arabidopsis roots, *AtAMT2;1* is induced by N starvation and weakly repressed by nitrate (Giehl et al., 2017), whereas the poplar homologue *PtrAMT2;1* is not regulated by N (Couturier et al., 2007), suggesting distinct regulation according to species.

The *ScAMT2;1* endogenous promoter drove the expression of GUS in Arabidopsis mainly at endodermal and pericycle cells in the innermost root tissue, with apparently more expression in roots subjected to ammonium compared with N deficiency. Previously, the activity of the Arabidopsis *AMT2;1* promoter was shown to become more confined to root endodermal and particularly pericycle cells when plants were exposed to high ammonium concentrations (Giehl et al., 2017). N deficiency, in turn, shifted the expression of *AtAMT2;1* towards the outer cells (Giehl et al., 2017). Our results with the heterologous expression of the *ScAMT2;1* promoter in Arabidopsis provide initial evidence that *ScAMT2;1* expression is concentrated on vascular and immediately surrounding tissues in roots and shoots. To confirm the predicted tissue-specific localization of *ScAMT2;1* in sugarcane, future studies based on *in situ* hybridization or transient or stable expression of *AMT2;1::GUS/GFP* directly in sugarcane will be necessary. Nevertheless, functional evaluation of sugarcane sequences, such as genes and regulatory regions, in a model plant provides initial inference before narrowing down to the target organism, such as described for sugarcane gene functional analysis (Wang et al., 2021; Chai et al., 2022).

Complementation of the ammonium uptake-defective yeast mutant (triple *mep*Δ) suggested that ScAMT2;1 is a functional NH_4_^+^ transporter, despite its lower substrate affinity than AtAMT1;1, similar to what had been previously described for AtAMT2;1 (Sohlenkamp et al., 2000). The expression of OsAMT2;1 in the same yeast mutant supported cell growth on media containing 5 mM NH_4_^+^ but not on 1 mM NH_4_^+^ (Suenaga et al., 2003). The poplar homologue PtrAMT2;1 complemented the triple *mep*Δ cells on 1 mM NH_4_^+^ (Couturier et al., 2007), while the homologues AtAMT2;1 (Sohlenkamp et al., 2000), LjAMT2;1 (Simon-Rosin et al., 2003), PbAMT2, and PbAMT3 (Li et al., 2016) restored the growth of the same yeast mutant strain cells on 0.5 mM NH_4_^+^ or even lower N concentrations, indicating a diverse biochemical transport capacity of the various AMT2 homologues.

Sugarcane ScAMT2;1 restored the growth of the triple *mep*Δ yeast in a pH-dependent manner, increasing activity consistently as the pH was raised from 5.0 to 6.0 while achieving the same growth of the negative control triple *mep*Δ+pDR196 at pH 7.5 (**Figure 3**), suggesting NH_3_ diffusion (Martinelle et al., 1996; Sohlenkamp et al., 2002). The apparent V_max_ of AtAMT2;1 determined in yeast also increased at higher pH values (Sohlenkamp et al., 2002). Ammonia is a weak base (pK_a_ 9.25), with more than 99% protonated at neutral external pH. Thus, elevating the pH from 5.0 to 7.5 increases the concentration of NH_3_ by 30-fold, while that of NH_4_^+^ remains almost constant (Sohlenkamp et al., 2002). The complementation of triple *mep*Δ by ScAMT2;1 at lower pH suggests that NH_4_^+^ rather than NH_3_ is the substrate for ScAMT2;1. Notably, as a common transport mechanism performed by AMT2 proteins, NH_4_^+^ appears to be deprotonated before transport, and NH_3_ permeates through the transporter pore (Khademi et al., 2004; Guether et al., 2009; Neuhäuser et al., 2009; Akgun and Khademi, 2011; Ariz et al., 2018). In yeast, ScAMT2;1 was unable to transport methylammonium (MeA), as triple *mep*Δ cells expressing ScAMT2;1 exhibited similar growth as cells expressing the empty vector (**Figure 3**). Previously, LjAMT2;1 and AtAMT2;1 were shown to be impermeable to MeA and to perform electroneutral transport of uncharged ammonia with a low transport capacity (Simon-Rosin et al., 2003; Neuhäuser et al., 2009). Thus, these three plant AMT2 homologues exhibit pH-dependent activity, being less active in acidic extracellular environments and displaying similar biochemical properties for NH_4_^+^ uptake and possible cotransport of NH_3_/H^+^ through the protein lumen (Neuhäuser et al., 2009). Certainly, expression analysis in *Xenopus* oocyte cells would be needed to validate this hypothesis.

The function of ScAMT2;1 was further supported by ectopic expression in the Arabidopsis *qko* mutant. Arabidopsis *qko* lines overexpressing *ScAMT2;1* accumulated more biomass under NH_4_^+^ nutrition as the only N source (**Figure 4**). Short-term influx analysis in *qko* complemented lines expressing *ScAMT2;1* allowed us to estimate a K_m_ equal to 90.17 µM and a V_max_ of 338.99 µmol h^-1^ g^-1^ root DW, suggesting that ScAMT2;1 contributes to high-affinity ammonium transport *in planta*. The estimated K_m_ value of ScAMT2;1 is higher than those determined for AtAMT1;1 and AtAMT2;1 *via* ^13^N-ammonium in yeast (22 and 21 µM at pH 6.1, respectively) (Sohlenkamp et al., 2002). However, despite the similar K_m_ values, the ammonium transport capacity of AtAMT2;1 was at least 10 times lower than that of AtAMT1;1 at a pH of 5.0 and 6.1. Nevertheless, at a pH of 7.5, the transport capacity of the two transporters appeared to be similar (Sohlenkamp et al., 2002).

Initial evidence for the possible function of ScAMT2;1 was obtained by heterologous expression of *ScAMT2;1* in Arabidopsis driven by one of its endogenous promoters (p2*ScAMT2;1*). Expression of p2*ScAMT2;1::ScAMT2;1* complemented the growth of *qko* plants under ammonium supply and significantly increased net ammonium influx only at high external N concentrations (**Figure 6**). These results and the localization of p2*ScAMT2;1* promoter activity (**Figure 5**) suggest that ScAMT2;1 mediates ammonium import from the apoplast, which is in agreement with previous studies with AtAMT2;1 (Sohlenkamp et al., 2002; Yuan et al., 2007; Neuhäuser et al., 2009). In Arabidopsis roots, AtAMT2;1 contributes to 10%–25% of the overall ammonium uptake rate at high external ammonium concentrations, whereas under N deficiency, AtAMT2;1 activity occurs in outer cell layers and supports root ammonium uptake capacity in the millimolar concentration range (Giehl et al., 2017).

Based on the localization of p2*ScAMT2;1* promoter activity, we hypothesized that ScAMT2;1 might be involved in root-to-shoot NH_4_^+^ translocation. Our findings indicated a contribution of ScAMT2;1 to shoot ammonium translocation only in fully ammonium- or nitrate-supplied plants (**Figure 7**). The increased root-to-shoot NH_4_^+^ translocation may have resulted from an increased ScAMT2;1-facilitated radial transport of ammonium towards the root vascular tissue, altering N partitioning between roots and shoots and impacting the N nutrition of the shoot. Although glutamine is the predominant organic N form translocated in the xylem of ammonium-supplied oilseed rape (Finnemann and Schjoerring, 1999) and *A. thaliana* plants (Lam et al., 1995), ammonium can represent 11% of the total N translocated in the xylem sap, reaching up to 18 mM in Arabidopsis vasculature (Giehl et al., 2017). Hence, increased root-to-shoot translocation of ammonium provides a stable supply of N to the shoots in response to high N availability. In Arabidopsis, a concerted function of AtAMT2;1 and GLN1;2 in roots is proposed to determine ammonium translocation and assimilation in response to high N supply (Giehl et al., 2017). Likewise, our results showing ScAMT2;1 activity in the innermost root cell suggest that this protein might also provide ammonium for the N assimilation pathway in sugarcane roots upon high ammonium supply. While GS1 activity has not been linked to yield gain or improved NUE in sugarcane genotypes (Robinson et al., 2007), ammonium is preferentially acquired by sugarcane roots (Robinson et al., 2011; Lima et al., 2022). Therefore, the critical role of ammonium translocation to shoots in response to plant nutritional status might significantly impact vegetative biomass in sugarcane plants. The mechanisms involved in root-to-shoot N transport, recycling, and remobilization are paramount for improving plant performance and NUE and can certainly decrease the need for fertilizers and strengthen sustainable sugarcane crop production. Our preliminary findings suggest that ScAMT2;1 might contribute to ammonium uptake in sugarcane roots in response to high external N availability in addition to presumably contributing to root-to-shoot ammonium translocation by facilitating its radial transport towards the vascular system, which may finally contribute to enhanced shoot growth under abundant N supply.

We conclude that ScAMT2;1 is a functional ammonium transporter as it was able to complement the defective Arabidopsis mutant and partially complement yeast. Estimation of K_m_ and V_max_ indicated ScAMT2;1 to be a high-affinity ammonium transporter. In sugarcane, *ScAMT2;1* is expressed in different organs, with the highest expression in roots induced by external nitrate (possibly lack of ammonium). When expressed in *A. thaliana*, *ScAMT2;1* promoter activity can be detected in the innermost cell layers of roots and the vasculature of leaves, and it can increase ammonium translocation from root to shoot. Our findings suggest that ScAMT2;1 might contribute to ammonium uptake in sugarcane roots in response to high external N availability and to probably contribute to root-to-shoot ammonium translocation by facilitating its radial transport towards the vascular system.

## Data availability statement

The original contributions presented in the study are included in the article/**Supplementary Material**. Further inquiries can be directed to the corresponding authors.

## Author contributions

AF and JL designed the research project. AK and RM performed experiments. JL, RM, and AK analyzed and interpreted the data. AK, RM, and NS performed bioinformatic analysis. NS helped to analyze/interpret the genomic data from BAC clones. MV contributed with analyses and protocols. AK and JL wrote the manuscript. RG critically reviewed the manuscript and helped with data interpretation. AF and JL supervised the project and experiments. All authors contributed to the article and approved the submitted version.

## Funding

This work was supported by FAPESP (The Sao Paulo Research Foundation) through Regular Research Grants (16/14669-8; 2013/15989-8), and fellowships to JL (2010/11313-1), and RM (2017/00460-2). Additional support came from CNPq (Brazilian National Council for Scientific and Technological Development) and CAPES (Coordination for the Improvement of Higher Education Personnel). AF is a recipient of a CNPq research fellowship (310645/2021-2).

## Acknowledgments

Seeds of *qko* were gently provided by Prof. Dr. Nicolaus von Wirén from the Leibniz Institute of Plant Genetics and Crop Plant Research.

## Conflict of interest

The authors declare that the research was conducted in the absence of any commercial or financial relationships that could be construed as a potential conflict of interest.

## Publisher’s note

All claims expressed in this article are solely those of the authors and do not necessarily represent those of their affiliated organizations, or those of the publisher, the editors and the reviewers. Any product that may be evaluated in this article, or claim that may be made by its manufacturer, is not guaranteed or endorsed by the publisher.
