# Supplementary material for: Functional characterization of the sugarcane (Saccharum spp.) ammonium transporter AMT2;1 suggests a role in ammonium root-to-shoot translocation
Source: Front Plant Sci. 2022 Nov 18;13:1039041. doi: 10.3389/fpls.2022.1039041 (PMC9716016; doi:10.3389/fpls.2022.1039041)
Supplement: Supplementary file 1 [file DataSheet_1.docx]

Supplementary Data


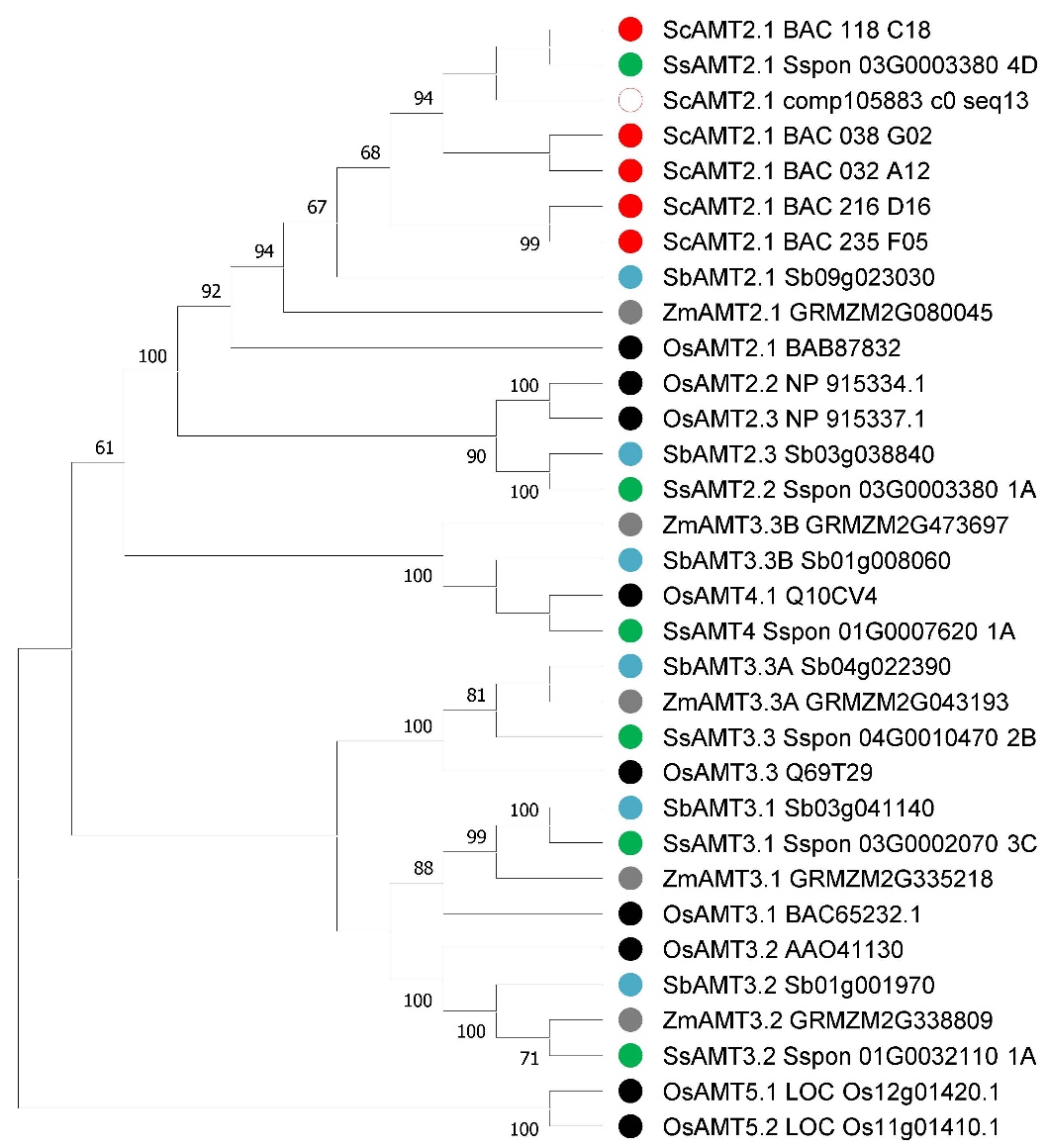


**Fig. S1.** Phylogenetic analysis based on the deduced amino acids of AMT2;1 sequences. The AMT2;1 sequences were identified in BAC clones (red circles), *S. spontaneum* (*SsAMT*, green circles; Wu et al*.*, 2021), and an assembled transcript derived from a RNA-seq from sugarcane roots (‘SP80-3280’) (empty red circles), together with members from rice (*OsAMT*), maize (*ZmAMT*), and sorghum (*SbAMT*). The tree was generated using Maximum Likelihood method and JTT matrix-based model using MEGA11 (Tamura et al., 2021). Bootstrap values (>70%) are indicated in each node.


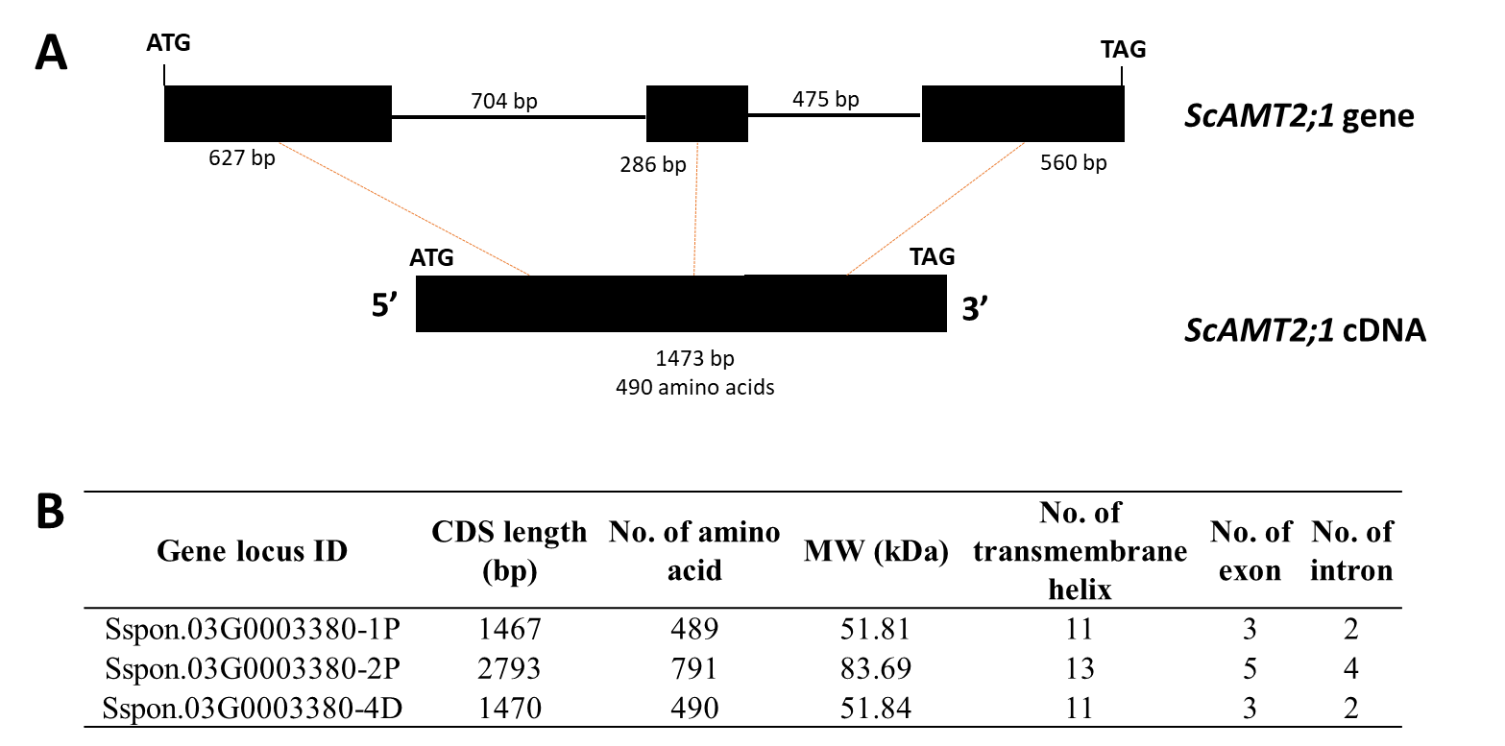


**Fig. S2. *ScAMT2;1* gene structure. (A)** Schematic structure of the ScAMT2;1 gene and cDNA from BAC 118_C18. Exons are indicated by dark boxes and lines represent introns. The exon and intron lengths (base pairs, bp) are indicated below and above the structures, respectively. **(B)** Characteristics of ScAMT2;1 in Saccharum spontaneum from chromosomes 7A (Sspon.03G0003380-1P); chromosomes 7B (Sspon.03G0003380-2P) and; chromosome 7D (Sspon.03G0003380-4D) (Wu et al*.*, 2021).


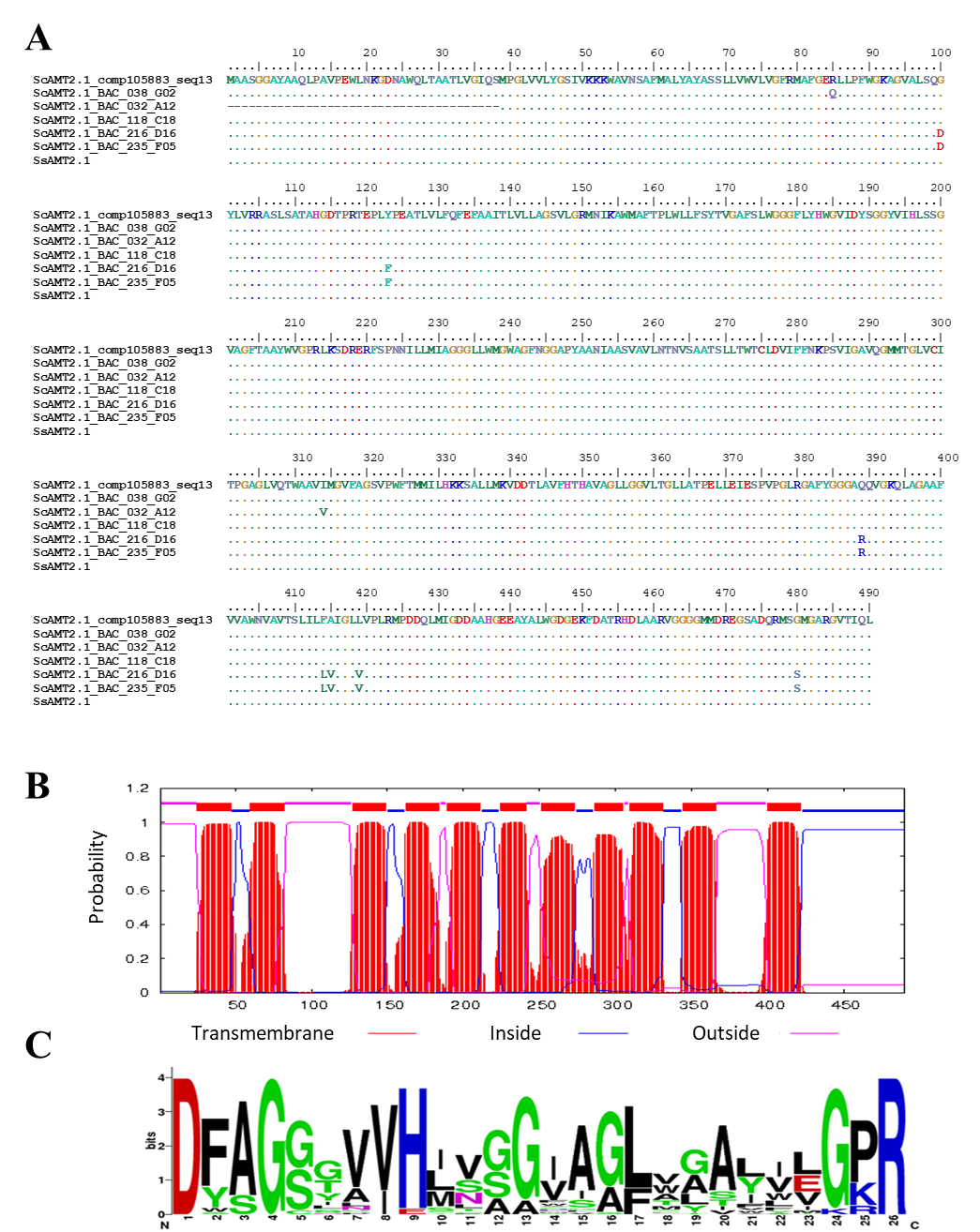


**Fig. S3.** *In silico* analyses of ScAMT2;1 protein features. **(A)** Alignment of the deduced amino acid sequence of ScAMT2;1 from a sugarcane RNA-seq ('SP80-3280'- comp105883; NCBI accession id OM966894), BAC clones (cultivar ‘R570’), and *S. spontaneum* (Sspon.03G0003380-4D; Wu et al., 2021). **(B)** Transmembrane topology profile of the ScAMT2;1 protein (BAC 118_C18) predicted by the TMHMM program. **(C)** WebLogo representation for the 26 amino acid signature motif conserved among 41 members of the MEP/AMT/Rh superfamily deposited at the PROSITE database (accession id PS01219).


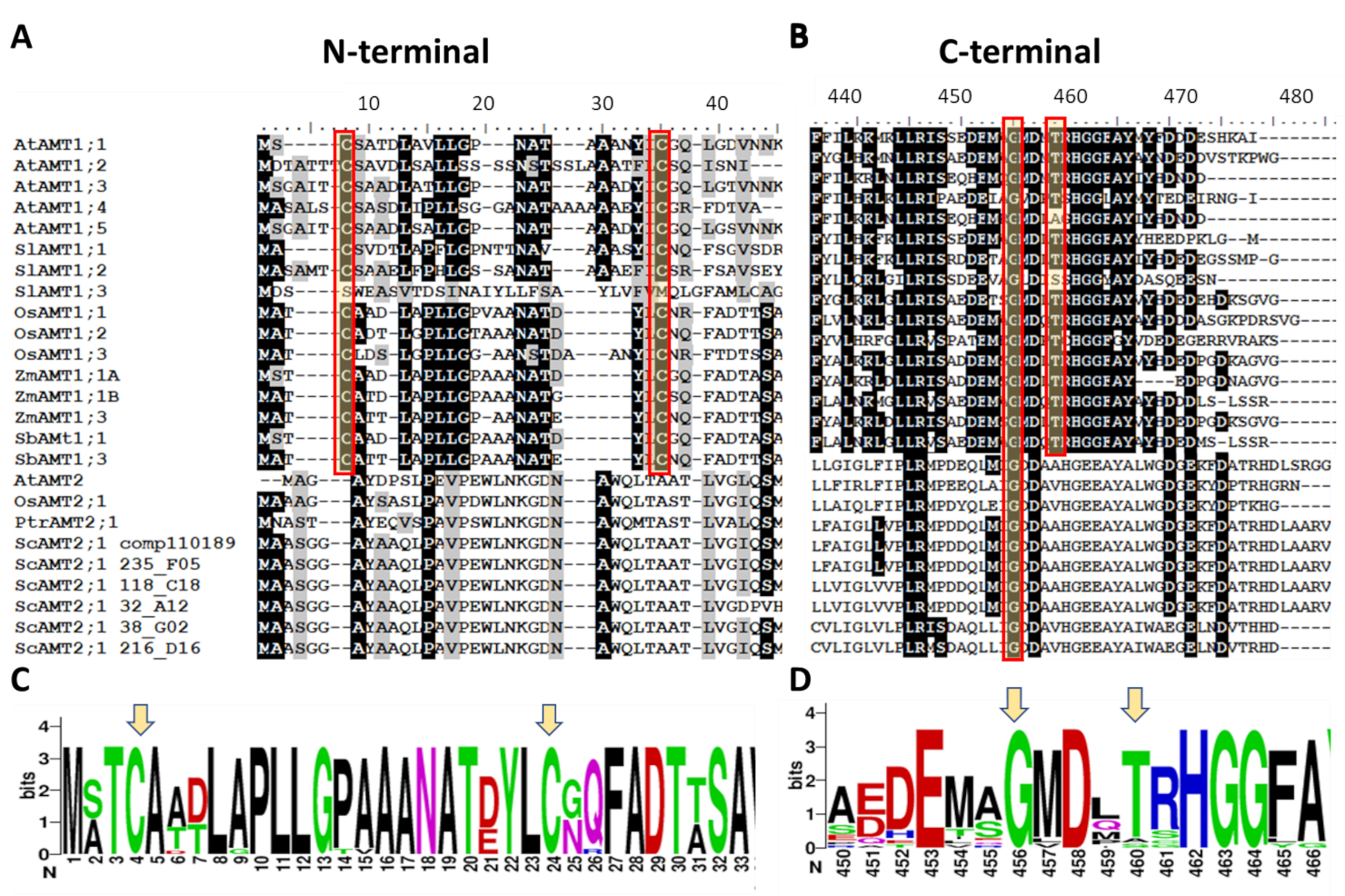


**Fig. S4.** N- and C-terminal amino acid sequences of plant ammonium transporters from the AMT1 and AMT2 subfamilies. **(A)** Alignment of 50 amino acids from the AMT N-terminal. Conserved AMT1 cysteines (corresponding to C3 and C27) are highlighted in red. **(B)** Alignment of 50 amino acids from the AMT C-terminal. Conserved glycine G456 and threonine T460 are highlighted in red. **(C)** WebLogo representation of conserved residues from the N-terminal sequence of AMT1 proteins, and **(D)** from the C-terminal sequence of AMT1 and AMT2 proteins. At: *A. thaliana*, Sl: *S. lycopersicum*, Os: *O. sativa*, Zm: Z. mays, Sb: *S. bicolor*, Sc: *Saccharum* spp*.,* and Pt: *P. trichocarpa*. The accession NCBI id numbers are: AtAMT1;1: P54144; AtAMT1;2: Q9ZPJ8; AtAMT1;3: Q9SQH9; AtAMT1;4: Q9SVT8; AtAMT1;5: Q9LK16; SlAMT1;1: P58905.1; SlAMT1;2: 004161.1; SlAMT1;3: Q9FVN0.1; OsAMT1;1: Q7XQ12.1; OsAMT1;2: Q6K9G1.1; OsAMT1;3: Q6K9G3.1; ZmAMT1;1A: AFW58821.1; ZmAMT1;1B: NP_001141280.1; ZmAMT1;3: NP_001130456.1; SbAMT1;1: XP_002446777.1; SbAMT1;3: XP_002452468.1; AtAMT2;1: Q9M6N7; OsAMT2;1: Q84KJ7; PtAMT2;1: B9HCZ0; LjAMT2;1: Q93X02; and ScAMT2,1 derived from BAC_032_A12; BAC_038_G02; BAC_118_C18; BAC_216_D16; and BAC_235_F05.


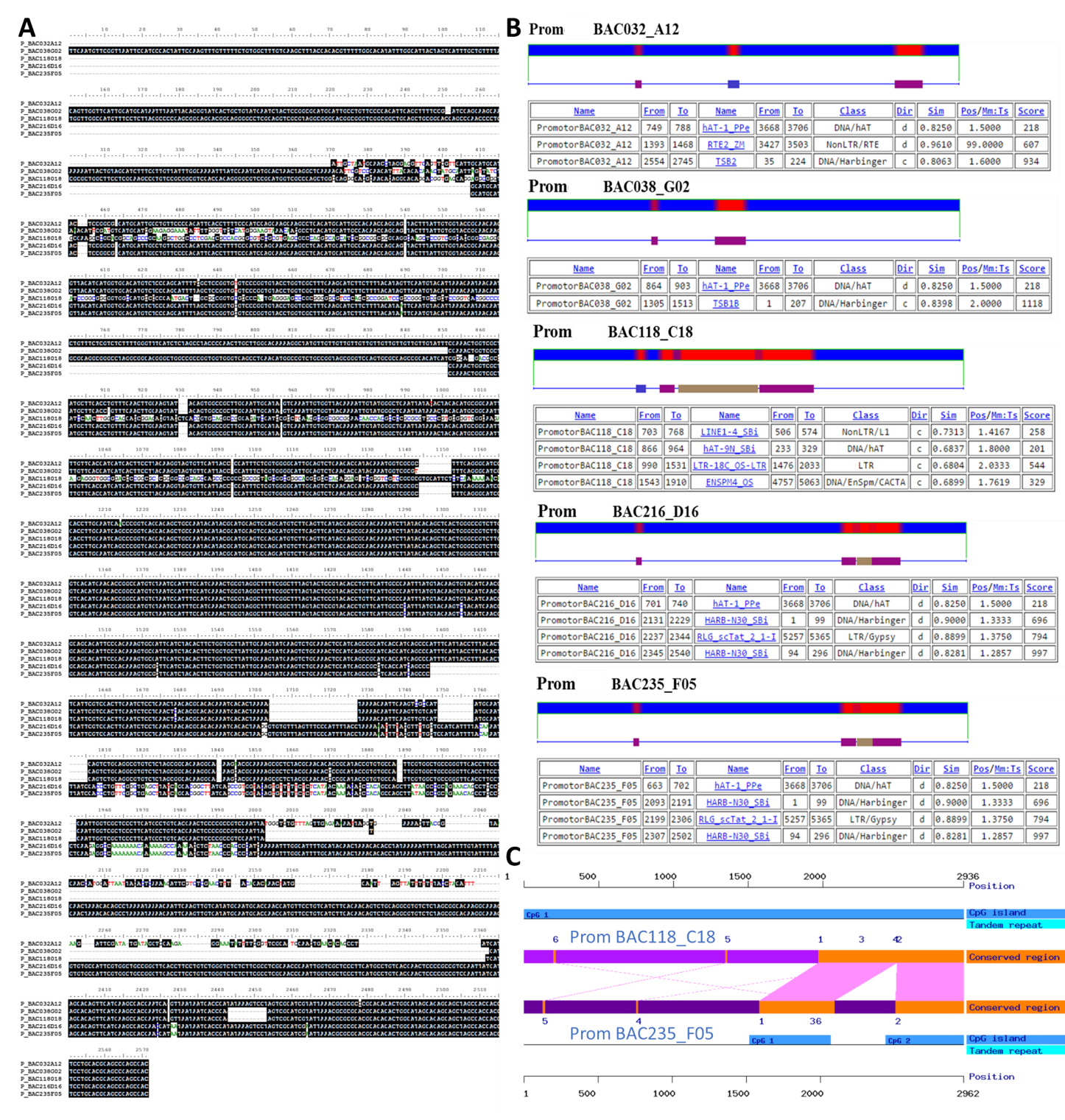


**Fig. S5.** *In silico* analyses of *ScAMT2;1* regulatory sequences. **(A)** Alignment of the *ScAMT2;1* regulatory region (approximately 3 kb upstream of translation start codon). **(B)** *In silico* analyses of regulatory sequences from *ScAMT2;1* for the presence of transposable elements. Transposable element name, classification, direction, and score were indicated by the Censor tool. **(C)** Synteny analysis of regulatory elements of *ScAMT2;1* regulatory region from BAC 118_C18, representing group 1, and BAC 235_F05, representing group 2, performed by the PlantPAN tool. TFBSs (transcription factor binding sites) are shown in pink, CpG islands in dark blue boxes, and tandem repeats in light blue boxes. Conserved motifs are show as orange traces linked together by purple dashes.


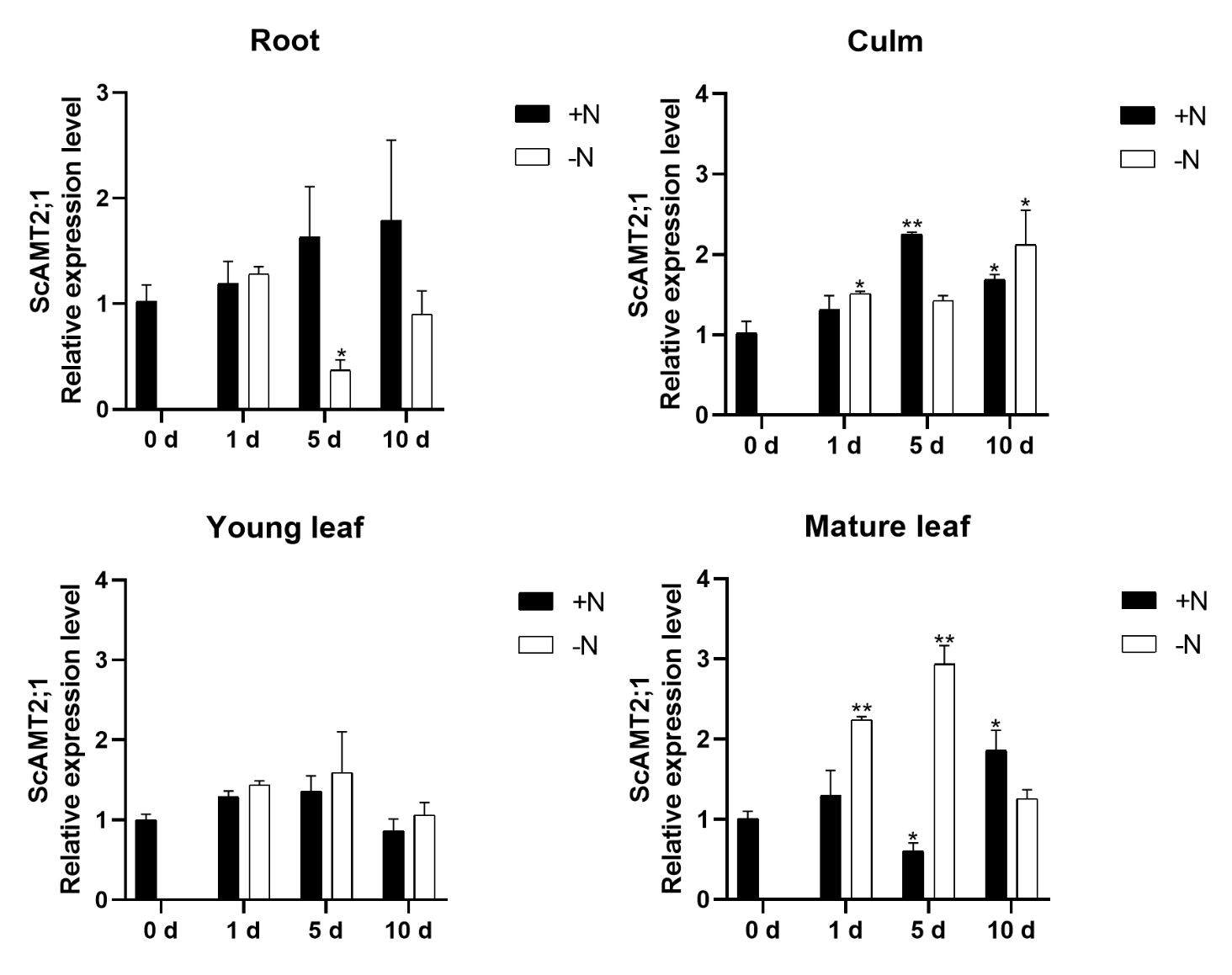


**Fig. S6.** *ScAMT2;1* expression analysis of sugarcane organs under N-free (-N) or high N (+N) conditions. Expression was estimated by RT-qPCR in root, culm, young leaf, and mature leaf of sugarcane subjected to high N (5 mM NH_4_NO_3_; +N) or no N (-N) for 1, 5, and 10 d. Bars indicate ± SE (*n*= 3 per sampled period). Gene expression was normalized to the respective tissues at 0 d (+N). *ScUBQ2* was used as reference gene. Asterisks represent significant differences between treatments and +N 0 d according to Student's *t* test (* p < 0.05 and ** p < 0.01). SE: standard error.


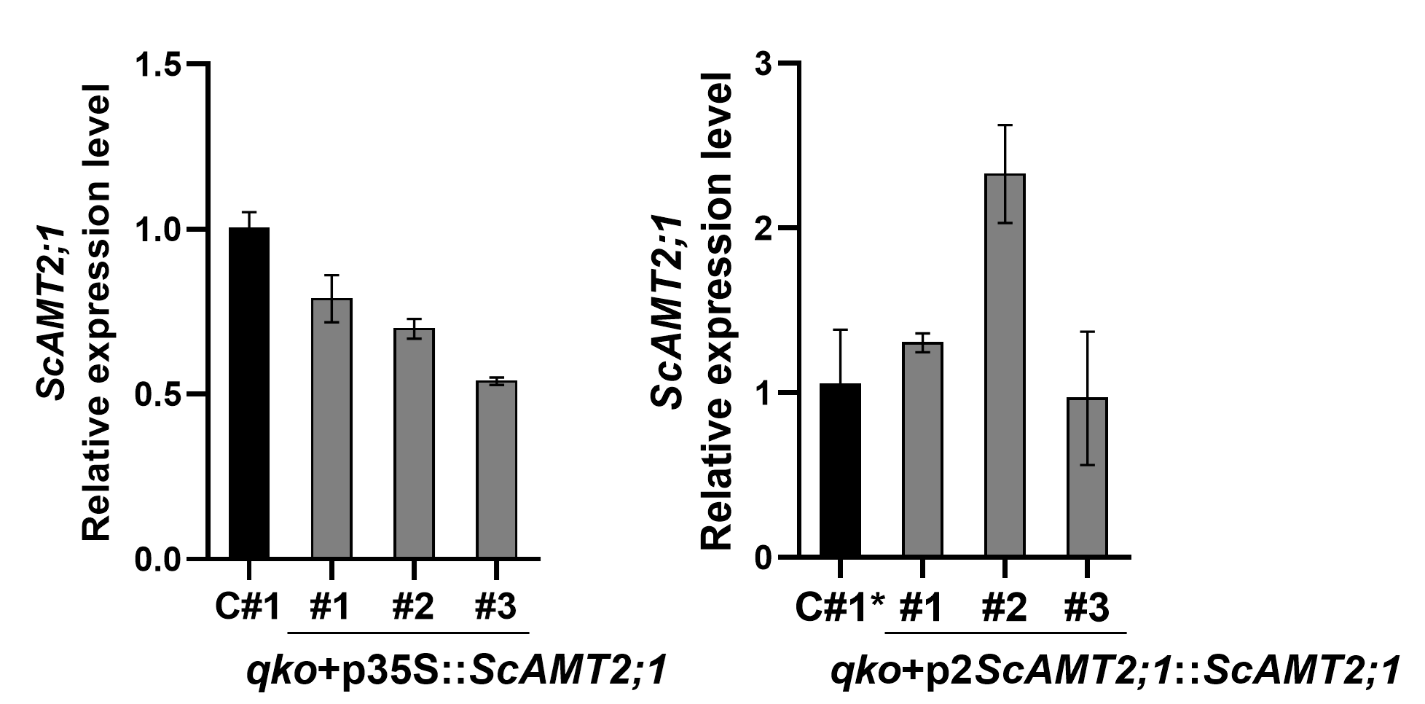


**Fig. S7.** *ScAMT2;1* relative expression in transgenic lines of Arabidopsis *qko* plants expressing *ScAMT2;1* under the regulation of the CaMV35S (p35S) promoter or *ScAMT2;1* endogenous promoter (p2 *ScAMT2;1*). The relative expression of *ScAMT2;1* were estimated by RT–qPCR using *AtUBQ2* as gene reference*.* Transgenic ‘Col-0’ plants were used for normalization of gene expression. C#1: Col-0+p35S::*ScATM2;1*; C#1*: Col-0+p2*ScAMT2;1*::*ScAMT2;1*. Bars indicate ± standard error (SE) (*n* = 3 independent pooled replicates).

**
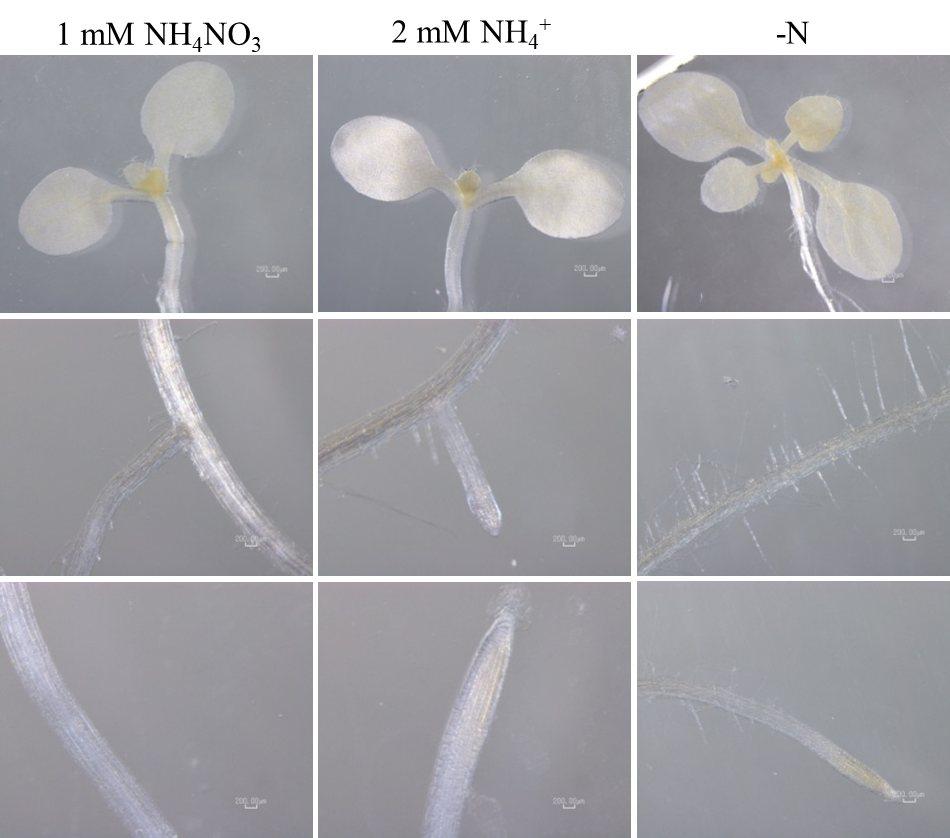
**

**Fig. S8.** Endogenous *ScAMT2;1* promoter 1 (BAC 118_C18) driving *uidA* (GUS) expression in Arabidopsis ‘Col-0’ plants. The transgenic lines (Col-0+p1*ScAMT2;1*::*uidA*) showed no GUS activity in shoots and roots when grown under 1 mM NH_4_NO_3_, 2 mM NH_4_^+^, or no N for up to 10 d. Bars = 200 μm.

**Table S1.** DNA sequence identification, primer sequences, amplicon size, and purpose.

| **ID** | **Primer sequence (5' → 3')** | **Amplicon size (bp)** | **Purpose** |
| --- | --- | --- | --- |
| *ScAMT2;1* gene F | ATGGCGGCGTCGGG | 1473 | Cloning - Plant |
| *ScAMT2;1* gene R | CTACAGCTGAATGGTGACGC |  |  |
| p1ScAMT2*;1* F | CACCGAACGACTGGCGACA | 2936 | Cloning - Plant |
| p1ScAMT2*;1* R | GTCGCTCGGCTCCGTGC |  |  |
| p2ScAMT2*;1* F | GGCTTAGCTGTTTGACTTGGTGC | 2962 | Cloning - Plant |
| p2ScAMT2*;1* R | GTCGCTCGGCTCCGTGC |  |  |
| p2+g-p2ScAMT2*;1* F | **GGGGACAAGTTTGTACAAAAAAGCAGGCTTA**GGCTTAGCTGTTTGACTTGGTGC | 1531  3020 | Cloning - Plant  Cloning - Plant |
| p2+g-p2ScAMT2*;1* R | **GGGGACAACTTTTGTATACAAAGTTGT**GTCGCTCGGCTCCGTGC |  |  |
| *AtAMT1;1* F | **GAATTC**ATGTCTTGCTCGGCCAC - *Eco* RI | 3020  1518 | Cloning - Plant  Cloning - Yeast |
| *AtAMT1;1* R | **CTCGAG**TCAAACCGGAGTAGGTG - *Xho* I |  |  |
| *ScAMT2;1* gene F -Y | **GAATTC**ATGGCGGCGTCGG - *Eco* RI | 1518  1485 | Cloning - Yeast  Cloning - Yeast |
| *ScAMT2;1* gen R- Y | **CTCGAG**CTACAGCTGAATGGTGAC - *Xho* I |  |  |
| q*ScAMT2;1* gene F | GGCAGCATCGTGAAGAAGAA | 1485  90 | Cloning - Yeast  RT-qPCR |
| q*ScAMT2;1* gene R | CACCAGCACCCACACCAG |  |  |
| *AtUbiq2* F | CCAAGATCCAGGACAAAGAAGGA | 90  222 | RT-qPCR  RT-qPCR |
| *AtUbiq2* R | TGGAGACGAGCATAACACTTGC |  |  |
| *ScUbiq2* F | CTTCTTCTGTCCCTCCGATG | 222  159 | RT-qPCR  RT-qPCR |
| *ScUbiq2* R | TCCAACCAAACTGCTGCTC |  |  |

p: promoter, g: gene, F- Forward, R- Reverse, Y: yeast, q: RT–qPCR. In bold - border sequences for multisite Gateway or restriction enzyme sites.

**Table S2.** Sequences cloned, entry and final vectors, final constructs, and target organism.

| **Sequence** | **Entry vector** | **Final vector** | **Construct** | **Organism** |
| --- | --- | --- | --- | --- |
| *ScAMT2;1* | PCR8 | pMDC32 | p35S::*ScAMT2;1* | Plant |
| p1*ScAMT2;1* | PCR8 | pMDC110 | p1ScAMT2 *;1*::*GFP* | Plant |
| p1*ScAMT2;1* | PCR8 | pMDC164 | p1ScAMT2*;1*::*GUS* | Plant |
| p2*ScAMT2;1* | PCR8 | pMDC110 | p2ScAMT2 *;1*::*GFP* | Plant |
| p2*ScAMT2;1* | PCR8 | pMDC164 | p2ScAMT2*;1*::*GUS* | Plant |
| p2*ScAMT2;1 + ScAMT2;1* | PDONR221 P1P5r (1) | pMDC99 | p2ScAMT2*;1*::*ScAMT2;1* | Plant |
|  | PDONR221 P5P2 (2) |  |  |  |
| *AtAMT1;1* | pGEM-T easy | pDR196 | pDR196::*AtAMT1;1* | Yeast |
| *ScAMT2;1* | pGEM-T easy | pDR196 | pDR196::*ScAMT2;1* | Yeast |

p: promoter.

# References

**Wu Z, Gao X, Zhang N, Feng X, Huang Y, Zeng Q, Wu J, Zhang J, Qi Y**. 2021. Genome-wide identification and transcriptional analysis of ammonium transporters in *Saccharum*. *Genomics* 113, 1671–1680.
